# Mapping light-harvesting and photoprotection responses in the Photosystem II antenna system of higher plants

**DOI:** 10.1093/plphys/kiaf588

**Published:** 2025-11-13

**Authors:** Zeno Guardini, Luca Dall’Osto, Rodrigo L Gomez, Roberto Caferri, Pierre Joliot, Roberto Bassi

**Affiliations:** Dipartimento di Biotecnologie, Università di Verona, Strada Le Grazie 15, Verona 37134, Italy; Dipartimento di Biotecnologie, Università di Verona, Strada Le Grazie 15, Verona 37134, Italy; Dipartimento di Biotecnologie, Università di Verona, Strada Le Grazie 15, Verona 37134, Italy; Dipartimento di Biotecnologie, Università di Verona, Strada Le Grazie 15, Verona 37134, Italy; Laboratoire de Biologie du Chloroplaste et Perception de la Lumière Chez les Microalgues, Institut de Biologie Physico-Chimique, CNRS UMR 7141, Sorbonne Université, Paris 75005, France; Dipartimento di Biotecnologie, Università di Verona, Strada Le Grazie 15, Verona 37134, Italy; Accademia Nazionale dei Lincei, Palazzo Corsini, Via della Lungara 10, Rome 00165, Italy; Anton Dohrn Experimental Marine Station, Villa Comunale, Naples 80121, Italy

## Abstract

Optimal photosynthetic performance of plants requires a balance between light energy capture and its use in downstream reactions. The process of light harvesting and its regulation are mediated by a complex array of antenna proteins, whose conservation throughout evolution suggests each complex serves a specific function in the diverse growth conditions in the natural subaerial environment. However, the specific roles of individual gene products in various antenna functions remain poorly understood. In this study, we investigated the Photosystem II antenna system by employing genome editing techniques targeted at subsets of *LHCB* genes and characterized *Arabidopsis thaliana* mutants missing specific components of the Photosystem II antenna: namely, the trimeric LHCII, the monomeric LHC, or both. The focus was on light-harvesting capabilities and photoprotective functions, which included exciton trapping cooperativity, non-photochemical quenching (NPQ) of excess excitation energy, and overall resistance to photoinhibition under excess irradiation, aiming to pinpoint the site(s) of the photoprotective responses. NPQ activity was present in all genotypes, indicating that each pigment-binding protein contributes to the overall quenching response. Within each antenna subgroup, NPQ activity did not rely on lutein, whereas zeaxanthin proved essential. Although trimeric LHCII provided the largest contribution to NPQ, the presence of monomeric Lhcbs was associated with enhanced Photosystem II phototolerance under excess light exposure. We conclude that the assembly of Photosystem II supercomplexes, including monomeric Lhcbs, is vital for maintaining PSII stability and functional integrity, playing a key role in preventing photoinhibition.

## Teaser

The functional topology of photoprotection responses shows that monomeric antennae enhance PSII connectivity and phototolerance, while the major antenna LHCII is crucial in thermal energy dissipation.

## Introduction

Photosynthesis converts sunlight into chemical energy (ATP) and reducing power (NADPH), which ultimately drives CO_2_ fixation into sugars. Photosystem (PS) II catalyzes the initial step of electron transport (ET) and consists of a dimeric core complex, which houses the reaction center (RC) responsible for water splitting and O_2_ evolution, and an antenna system, made of light-harvesting complexes (LHC), which enhances photon capture (Wei et al. 2016). In the PSII supercomplex, 3 monomeric subunits (Lhcb4, Lhcb5, and Lhcb6) bridge excitation energy transfer between the (Lhcb1-3 peripheral trimeric LHCII) and the PSII core (Su et al. 2017). Trimeric LHCII is the most abundant pigment-binding protein on Earth, coordinating chlorophyll (Chl) *a*, *b*, and xanthophyll ligands such as neoxanthin, violaxanthin (Vio), and lutein (Lut) (Liu et al. 2004). Under limitant irradiance, LHCII enhances photon interception (Fuciman et al. 2012). However, under excess light (EL) conditions, excitation energy buildup outcompetes energy use by downstream metabolic reaction, thus increasing the probability that unquenched singlet excited states of Chl (^1^Chl*) transition to triplet state (^3^Chl*). This state reacts with O_2_, releasing singlet oxygen (^1^O_2_) and causing photoinhibition (Fischer et al. 2012). Fast-activated mechanisms that dissipate excess ^1^Chl* as heat, preventing oxidative damage, are collectively known as non-photochemical quenching (NPQ) (reviewed by Bassi and Dall’Osto 2021). Without additional stress factors exacerbating the response, NPQ can convert up to 70% of absorbed photons into heat, which is detected as a light-dependent decline in Chl fluorescence (Ishida et al. 2014), significantly reducing PSII quantum yield. The overall NPQ process can be dissected into several components (Horton et al. 1996; Cazzaniga et al. 2013; Malnoë 2018). The fastest component, qE (energy quenching), is activated within a few seconds and requires (i) lumen acidification, (ii) protonation of lumen-exposed acidic residues of the pH-sensor PsbS (Li et al. 2004), and (iii) the conversion of Vio to zeaxanthin (Zea) via the xanthophyll cycle (Niyogi et al. 2001).

The exact location of the quenching center(s) has long been investigated (for a review, see Ruban and Wilson 2021). Long-living excited states reside on Chls, implying that quenching sites are found on Chl-binding proteins. Since PsbS is a chromophore-less protein (Fan et al. 2015), qE reaction in higher plants likely occurs within one of the interacting pigment-binding subunits of PSII (Havaux et al. 2007). The Lhcb proteins constituting the PSII antenna are the most likely candidates, as they (i) bind Lut and Zea, which are required for NPQ activity (Niyogi et al. 2001), and (ii) interact with PsbS (Gerotto et al. 2015). Furthermore, the quenching amplitude was proportional to the Lhcb/RC ratio (Havaux et al. 2007; Ware et al. 2015a). The PSII antenna system is encoded by 14 homologous genes in *Arabidopsis thaliana* (Jansson 1999), making localization of quenching site(s) complex. Fluorescence quenching can be induced in vitro by aggregation in detergent-purified trimeric LHCII (Horton et al. 1996), or by adding PsbS (Wilk et al. 2013), suggesting that NPQ in vivo may also be triggered by aggregation within thylakoids (Ware et al. 2015b). Monomeric Lhcbs have also been shown to undergo quenching both in vitro and in vivo, consistent with their high affinity for Zea (Morosinotto et al. 2002). This suggests that 2 quenching sites (Q1 and Q2), located in trimeric LHCII and in the monomeric Lhcbs, respectively, contribute to NPQ activity (Betterle et al. 2009; Holzwarth et al. 2009). Reverse genetics allows for the assessment of biological activities involving multiple components. Indeed, NPQ kinetics were altered in the *koLhcb4* mutant (de Bianchi et al. 2011) and in the *NoM* mutant, which lacks all monomeric Lhcbs (Dall’Osto et al. 2017), suggesting a catalytic role for these subunits. A pigment cluster within Lhcb4, consisting of Chl pair a603-a609 and the Car at site L2 was identified as essential for quenching in vivo (Guardini et al. 2020). The *NoM* mutant displayed residual qE activity, although slowly activated (Dall’Osto et al. 2017), implying that LHCII hosts additional PsbS-dependent quenching sites (Pietrzykowska et al. 2014).

In addition to ^1^Chl* quenching, LHCs are involved in several photoprotective mechanisms. They actively quench ^3^Chl* through carotenoid triplet (^3^Car*) formation (Santabarbara et al. 2002), thereby preventing the formation of ROS. Zea plays a significant role in these photoprotective processes (Havaux and Niyogi 1999).

Noteworthy, the photoprotective effect of Zea is significantly enhanced when it binds to LHC proteins following the exchange with Vio (Morosinotto et al. 2002). Hence, alongside ROS scavenging in the lipid phase (Havaux et al. 2007) and the augmentation of qE, there exists a third photoprotective mechanism attributed specifically to the LHC-bound Zea pool, which reduces the yield of harmful ^3^Chl* (Dall’Osto et al. 2012).

In this study, we employed genome editing technology to thoroughly dissect the LHC components of the PSII antenna. Our objectives were to map: (i) excitation energy diffusion and trapping within PSII, (ii) thermal energy dissipation, and (iii) overall resistance to photoinhibition under excess irradiation. Results showed that monomeric complexes strongly contributed to efficient energy transfer and promoted PSII connectivity and were associated with the highest PSII phototolerance. Conversely, trimeric LHCII contributed most significantly to thermal energy dissipation. However, NPQ activity was observed across all genotypes, indicating that both monomeric and trimeric LHCs are involved in catalyzing quenching responses.

## Results

### *koLHCII* and *koLhcb* are *Arabidopsis* mutants lacking, respectively, Lhcb1-3 and Lhcb1-6 PSII antenna subunits

The construction of *A. thaliana* genotypes with altered Lhcb protein composition was based on 2 knock-out (ko) lines: *koLhcb3*, missing the Lhcb3 component of the major LHCII complex, and the *NoM koLhcb3* genotype, devoid of the *Lhcb4-6* genes encoding the monomeric antennae Lhcb4 (CP29), Lhcb5 (CP26), and Lhcb6 (CP24) (Dall’Osto et al. 2017), in addition to Lhcb3. A CRISPR-Cas9 mutagenesis strategy was used to target each of the 5 *Lhcb1* and 3 *Lhcb2* genes (Supplementary Table S1). This approach led to the creation of either the *koLHCII* line (lacking all LHCII but retaining monomeric Lhcb4-6 proteins), or the *koLhcb* line, missing both monomeric and trimeric LHCs of PSII. The above genotypes were further edited to knock out PSBS, LCYE (lycopene-ε-cyclase, Lut biosynthesis), or VDE (violaxanthin de-epoxidase, Zea biosynthesis), in order to map the contribution of these components to NPQ activity. The study also included mutants *NoM* (lacking all monomeric Lhcbs), *ch1* (unable to accumulate Chl *b*), *ch1 koLhcb5* (devoid of both Chl *b* and Lhcb5 minor antenna), and *chl1 koLhcb5 npq4* (lacking Chl *b*, Lhcb5, and PsbS), serving as control genotypes with compromised light-harvesting systems.

**Figure 1.**
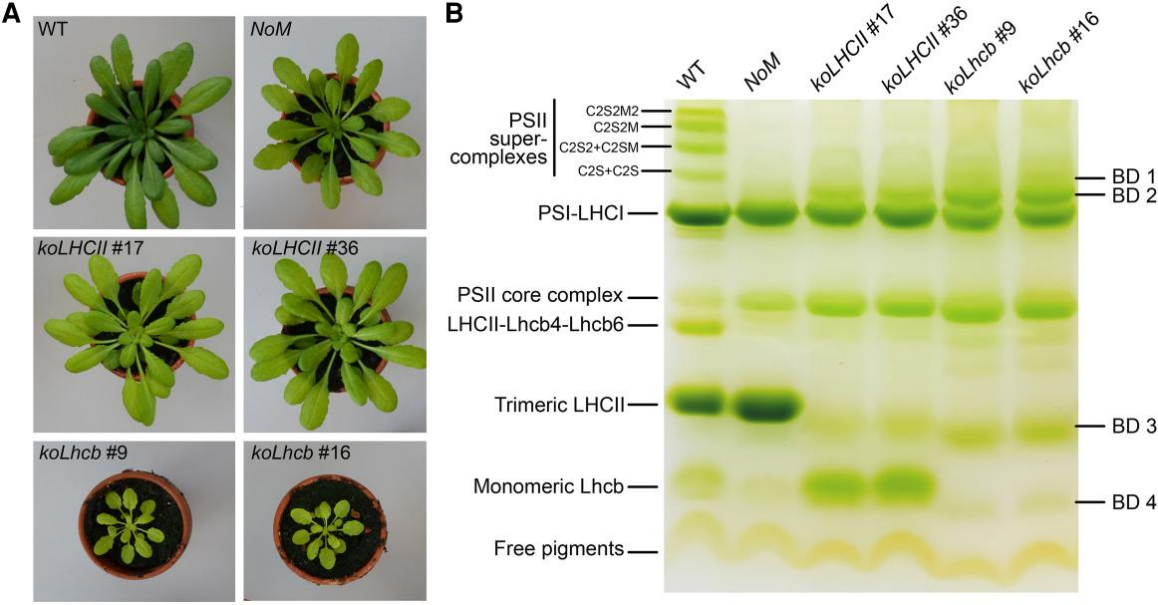
Phenotype of wild-type and mutant plants. **A)** Plants were grown for 6 weeks under conditions of 150 *μ*mol photons m^−2^ s^−1^, 23/19 °C (day/night), with an 8/16 h light/dark cycle. **B)** Non-denaturing Deriphat-PAGE of thylakoids solubilized with 0.8% α-DM, revealing the pigment-protein complexes of wild type, *NoM*, *koLHCII*, and *koLhcb* lines. Each lane contained thylakoid proteins corresponding to 35 *µ*g of Chl. The composition of the major bands is indicated based on previous reports, while the composition of bands BD1-BD4 was determined from absorption spectra and SDS-PAGE (see Supplementary Fig. S3).

When grown under controlled conditions (150 *μ*mol photons m^−2^ s^−1^, 23/19 °C, 8/16 h day/night), *NoM* and *koLHCII* exhibited a similar growth rate, though significantly impaired compared to wild type plants. The *koLhcb* lines showed a far stronger reduction in growth (Fig. 1A and Table 1), comparable to that of the *ch1 koLhcb5* and *ch1 koLhcb5 npq4* mutants (Supplementary Fig. S1; Table 1). The size of the *ch1* plants was intermediate between *koLHCII* and *ch1 koLhcb5*, suggesting that Lhcb5 plays a significant role in enhancing light-harvesting function, even in the absence of Chl *b* in this genotype (Supplementary Fig. S1).

**Table 1.** Pigment content of leaves from Arabidopsis WT, npq4, ch1, and LHC mutant lines

Genotypes	Chl *a*/*b*	Chl/Car	µg Chl cm^−2^
WT	3.51 ± 0.09^a^	3.69 ± 0.21^a^	19.4 ± 0.5^a^
*npq4*	3.40 ± 0.02^a^	3.66 ± 0.13^a^	21.1 ± 1.4^a^
*NoM*	3.28 ± 0.05^a^	3.45 ± 0.09^a,c^	15.3 ± 0.74^b^
*koLhcb #16*	6.14 ± 0.22^b^	3.09 ± 0.08^d^	6.10 ± 0.76^d^
*koLhcb #9*	6.28 ± 0.15^b^	3.12 ± 0.05^d^	5.68 ± 0.15^d^
*koLHCII #17*	5.23 ± 0.11^c^	3.37 ± 0.06^c^	11.46 ± 0.55^c^
*koLHCII #36*	5.15 ± 0.10^c^	3.34 ± 0.02^c^	11.03 ± 0.58^c^

Values were measured in homozygous F3 plants. The Chl/Car represents the molar ratio between chlorophylls (*a* + *b*) and carotenoids. Fresh weight refers to plants grown for 6 weeks under control conditions (150 *μ*mol photons m^−2^ s^−1^, 23 °C, 70% humidity, and a day/night cycle of 8/16 h). All data are expressed as mean ± Sd, *n* = 5 biologically independent plants. Values marked with different letters (a–d) significantly differ within the column (ANOVA, followed by Tukey's post hoc test at a significance level of *P* < 0.05). The experiments were independently repeated twice, yielding similar results.

To determine if the observed growth effect was due to changes in the abundance of supercomplexes within the photosynthetic membranes, the levels of selected thylakoid proteins were measured using immunotitration (Supplementary Fig. S2). *koLHCII* and *koLhcb* lacked Lhcb1-3 and Lhcb1-6 subunits, respectively, while Lhcb5 was present in substantial amounts in *ch1* (170% respect to wild type; Supplementary Fig. S2B). The PSI (PsaA) content was reduced to a similar extent across all mutants depleted of LHCs, consistent with evidence that a high PQ/PQH_2_ ratio decreases the transcription of the PSI core subunit PsaB (Tullberg et al. 2000). However, the levels of ATPase β-subunit and Cytochrome *f* were similar across all genotypes. The supramolecular organization of pigment-protein complexes in antenna mutants was investigated using non-denaturing Deriphat-PAGE upon mild-solubilization with (0.8%) α-DM (Fig. 1B). The *NoM* genotype lacked PSII supercomplexes and over-accumulated the major trimeric antenna LHCII. In contrast, the trimeric LHCII band was absent in both *koLHCII* and *koLhcb* genotypes, which also lacked high molecular weight supercomplexes except for 2 bands, BD1 and BD2, migrating just above PSI-LHCI. A faint green band (BD3) was observed migrating slightly below trimeric LHCII in both *koLHCII* and *koLhcb*, and a fourth band (BD4) migrated similarly to the monomeric Lhcbs. Biochemical and spectroscopic analyses revealed BD1 and BD2 to comprise PSI-LHCI complexes with different LHC complements, BD3 contained dimeric LHCI subunits and/or assembly of Lhcb4-Lhcb6 detached from PSII complexes, depending on genotypes; BD4 consisted of monomeric Lhcbs in *koLHCII* and monomeric LHCIs in *koLhcb* (Supplementary Fig. S3). A more detailed biochemical analysis of the LHC composition in *koLHCII* plants provided evidence to exclude both compensatory upregulation and oligomeric organization of the minor LHCs (Supplementary Fig. S4).

After assessing how the loss of specific LHC subunits impacts antenna organization around PSII, we examined the effects of these depletions on key photosynthetic functions: (i) excitation energy diffusion and trapping in PSII, (ii) PSII operational efficiency during photosynthesis, (iii) capacity for feedback de-excitation, and (iv) sensitivity to EL treatments.

### LHC depletion impacts PSII excitation energy diffusion and trapping

**Figure 2.**
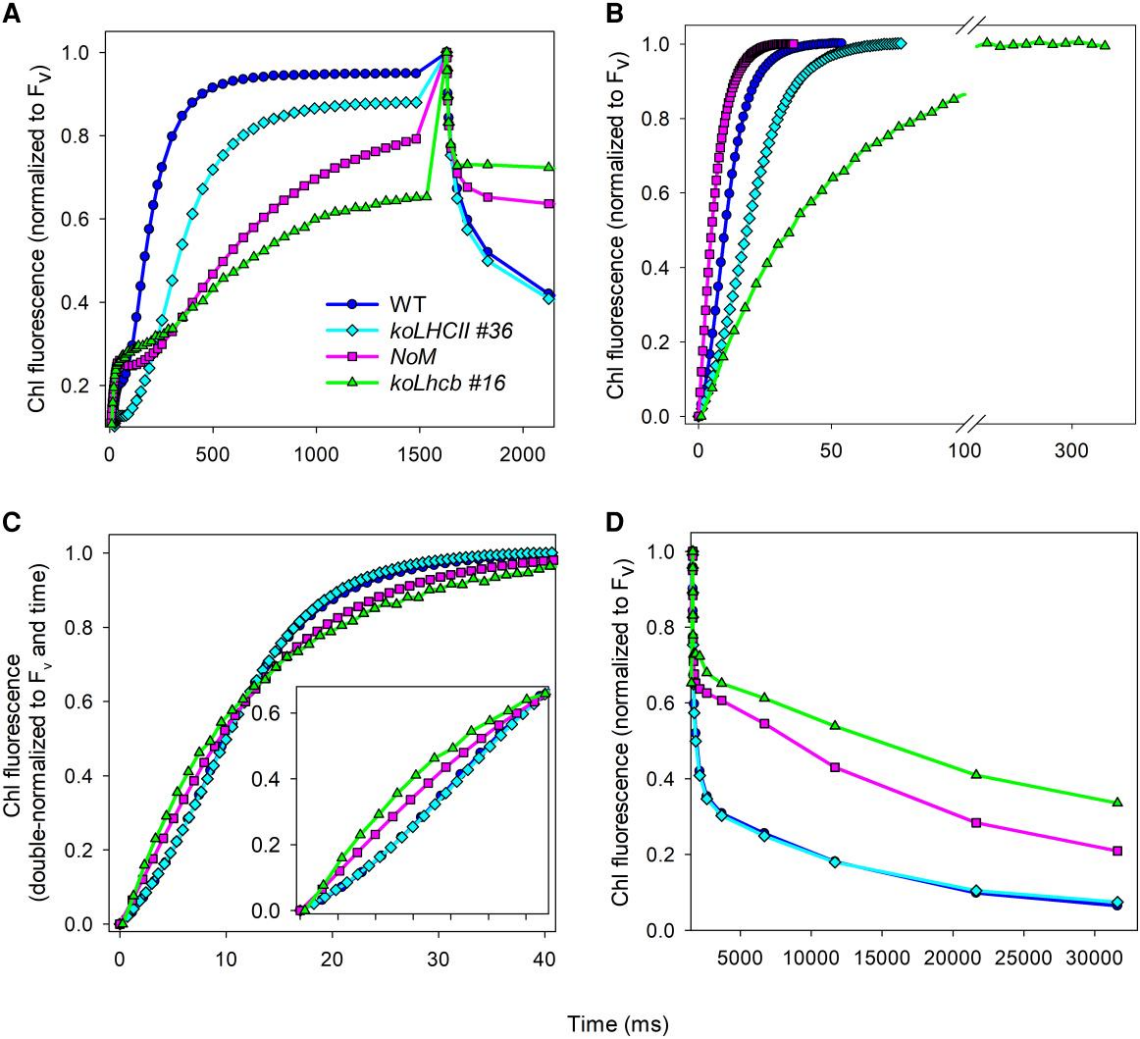
PSII Chl fluorescence kinetics. Fluorescence rise was measured at RT in dark-adapted leaves, using weak light (7 *µ*mol photons m^−2^ s^−1^) in both untreated **A** and **D**) and DCMU-treated leaves **B** and **C**). The maximum fluorescence yield was measured 100 µs after a pulse of saturating light. The experimental fluorescence curves were normalized to their corresponding F_v_ values **A, B,** and **D**), or double-normalized to F_v_ and time, so that the curves intersect at the same point where fluorescence = 0.6, using WT as reference **C)**. Each trace represents the average of 3 independent experiments.

To investigate the role of distinct Lhcbs in the transfer and trapping of excitation energy by PSII RC, we measured fluorescence kinetics at the onset of illumination, both with and without DCMU at RT (Fig. 2, A and B; Supplementary Table S2). In the presence of DCMU, the normalized kinetics exhibited a precise sigmoidal shape in the wild type and *koLHCII*, whereas the *koLhcb* and *NoM* strains showed patterns closer to an exponential function (Fig. 2B). The non-exponential behavior reflects a fluorescence yield not linearly related to the Q_A_ concentration (Genty et al. 1989; Lavergne and Trissl 1995), due to excitation transfer within domains including multiple PSII RCs. As fluorescence is linearly related to PSII photochemical rate (Bennoun and Li 1973), we plotted the traces from panel 2B after normalizing the time scales to the functional antenna size (Fig. 2C). This normalization factor (0.55, 1.9, and 0.25 for the *koLHCII*, *NoM*, and *koLhcb*, respectively) ensured the same area above the curves, effectively normalizing to the same RC concentration and providing an accurate estimate of the optical cross-section in mutants compared to the wild type. We observed a similar slowdown of the fluorescence rise in the *koLHCII* compared to the wild type in the absence (Fig. 2A) and presence (Fig. 2, B and C) of DCMU. This result suggests that the rate of reduction of PSII electron acceptors was decreased in the same proportion as the optical cross-section (i.e. by ∼2.1-fold). Additionally, the ratio between the size of the PQ pool and the number of PSII RCs was equal in both strains.

The light-harvesting and ET activities were studied by analyzing the Chl fluorescence decay kinetics after a pulse of saturating light on the wild type and the *koLHCII*, without DCMU (Fig. 2D). These kinetics reflect the oxidation of Q_A_ after its complete reduction by the light pulse, depending on the degree of connectivity, which defines the non-linear relationship between Q_A_ concentration and Chl fluorescence yield. Decay kinetics also depend on the apparent equilibrium constant of electron transfer between Q_A_ and the PQ pool. During illumination and dark relaxation, the PQ pool is not in thermodynamic equilibrium due to restricted PQ diffusion within small domains that include an average of 3 to 4 RCs (Joliot et al. 1992; Lavergne et al. 1992). The fluorescence recovery kinetics were similar in wild type and *koLHCII* leaves, while *NoM* and *koLhcb* showed much slower relaxation (Fig. 2D).

### Excitation energy regulation processes are compartmentalized in the PSII antenna system

PSII function during photosynthesis was assessed by analyzing Chl fluorescence. The ratio of variable to maximum fluorescence (F_v_/F_m_) generally showed a decrease linked to the reduction of the biochemical antenna (Table 2). Such a decrease in F_v_/F_m_ can result from various factors, such as diminished photochemical yield of the PSII RC, changes in the macro-organization of the thylakoid membrane (Ilikova et al. 2021), or alterations in the relative contributions of PSI and/or detached LHC to the F_0_ and F_v_ values (Franck et al. 2002).The fraction of PSII in the open state (qL; see Supplementary Fig. S5) was higher in *koLHCII* compared to wild type under irradiance levels below 500 *µ*mol photons m^−2^ s^−1^, and even higher in all other LHC-depleted mutants (*koLhcb*, *ch1 koLhcb5*) at all light intensities tested. This suggests that PSI vs. PSII excitation imbalance occurred in LHC-depleted lines.

**Table 2.** Chl fluorescence induction parameters in leaves from wild-type and mutant plants

Genotypes	F_0_/Chl (norm. to WT)	F_v_/F_m_	1/t_2/3_ (·10^−3^, ms^−1^)	*J*
WT	1.0 ± 0.08^a^	0.82 ± 0.01^a^	5.76 ± 0.42^a^	2.05 ± 0.24^a^
*NoM*	3.23 ± 0.26^b^	0.61 ± 0.01^b^	10.70 ± 0.98^b^	0.79 ± 0.13^b^
*koLhcb #16*	5.46 ± 0.88^c^	0.54 ± 0.03^d^	1.74 ± 0.18^d^	0.60 ± 0.39^b^
*koLhcb #9*	5.18 ± 0.99^c^	0.50 ± 0.02^d^	1.95 ± 0.18^d^	0.58 ± 0.25^b^
*koLHCII #17*	2.13 ± 0.28^d^	0.76 ± 0.01^c^	2.47 ± 0.31^c^	1.76 ± 0.07^a^
*koLHCII #36*	2.18 ± 0.24^d^	0.76 ± 0.01^c^	2.66 ± 0.26^c^	1.71 ± 0.10^a^

F_0_ (minimal Chl fluorescence of PSII) values were recorded using the Dual-PAM and normalized to the corresponding Chl content per unit leaf surface. The PSII functional antenna size (1/t_2/3_) and the connectivity parameter *J* were determined from fast Chl fluorescence induction in DCMU-treated leaves, measured using green light (7 *µ*mol photons m^−2^ s^−1^). All data are expressed as mean ± Sd, *n* = 5 biologically independent plants. Values marked with different letters (a–d) significantly differ within the column (ANOVA, followed by Tukey's post hoc test at a significance level of *P* < 0.05). The experiments were repeated independently twice, with similar results.

Chl fluorescence yield (Φ_F_) indicates how excitation energy is partitioned during photosynthesis (Kramer et al. 2004; Ahn et al. 2008). The quantum yield of PSII photochemistry (Φ_II_) in dark-adapted leaves was lowest in *koLhcb*, and intermediate in *koLHCII* and *ch1 koLhcb5* compared to wild type. Φ_II_ rapidly declined with increasing irradiance, eventually reaching similar values across all genotypes (Supplementary Fig. S5, D to F).

The yield of dissipation via active downregulation (Φ_NPQ_) increased with light intensity, reaching 0.6 in wild type and *NoM*, 0.4-0.45 in *koLHCII* and *ch1 koLhcb5*, and remained very low, though still active (0.2) in *koLhcb*. In contrast, the yield of other non-photochemical, unregulated, energy losses (Φ_NO_) remained steady at 0.28 across light intensities above 200 *µ*mol photons m^−2^ s^−1^ in wild-type leaves (Supplementary Fig. S5, G to I). However, it increased to 0.6 in *koLHCII* and *ch1 koLhcb5*, and to 0.8 in *koLhcb*, indicating significant limitations in compensatory changes in Φ_II_ and Φ_NPQ_ at higher irradiance levels in mutants with severely affected PSII antennae.

The rise in Φ_NO_ in dark-adapted leaves of LHC-depleted genotypes led to an increase of F_0_, likely due to reduced efficiency in driving photochemistry or an increased contribution of PSI-associated Chl to the overall fluorescence compared to wild type (Franck et al. 2002) (Supplementary Fig. S5, J to L).

### LHC-depleted mutants have a reduced capacity for feedback de-excitation

**Figure 3.**
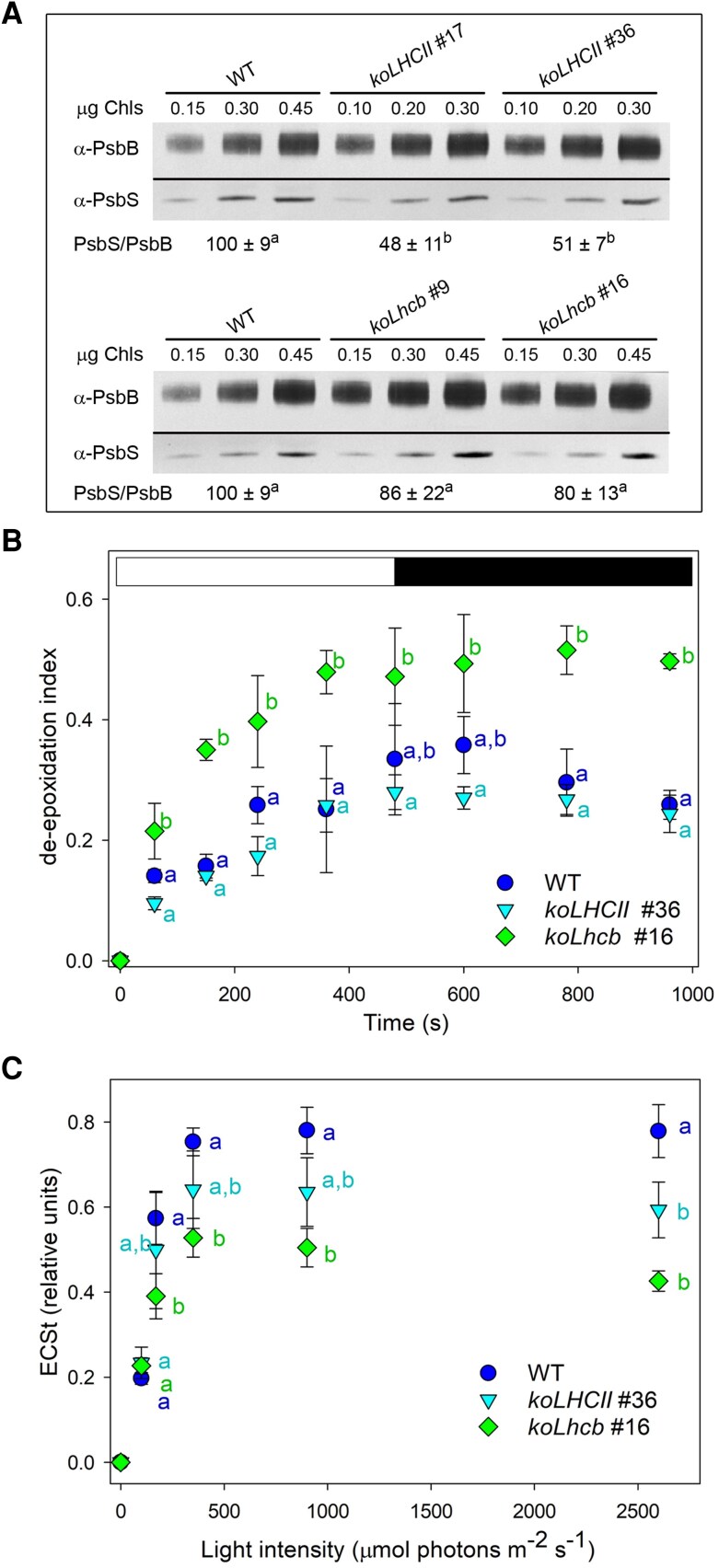
Analysis of the main factors controlling NPQ amplitude and kinetic. **A)** Immunotitration of leaf extracts was performed using α-PsbS and α-CP47 antibodies. PsbS content was normalized to the PSII core (PsbB content) and expressed relative to the corresponding wild-type value. Data are reported as mean ± Sd, *n* = 3 biologically independent samples. Values marked with different letters significantly differ (ANOVA followed by Tukey's post hoc test at a significance level of *P* < 0.05). **B)** Time course of Vio de-epoxidation in wild-type and mutant plants. Dark-adapted leaf discs were illuminated with 1000 *µ*mol photons m^−2^ s^−1^ (white actinic light) for 8 min, followed by 8 min of dark recovery. At different time points, leaf discs were frozen in liquid nitrogen, and pigments were extracted for HPLC analysis. White and black bars represent light and dark periods, respectively. **C)** ECS_t_ vs. light intensity, measured at steady-state photosynthesis, estimates the light-driven pmf across the thylakoids. All data are expressed as mean ± Sd (*n* = 5). In **B)** and **C)**, values marked with different letters at a given time point significantly differ (ANOVA followed by Tukey's post hoc test at a significance level of *P* < 0.05).

NPQ is essential for preventing excessive energy buildup in PSII. Since most quenching activity was associated to the PSII antenna system (Horton and Ruban 2004), we focused on the elucidation of the mechanistic role of Lhcb subunits in promoting NPQ activity. First, we evaluated the abundance of components involved in NPQ, including the lumenal pH sensor PsbS, the extent of thylakoid lumen acidification, and the xanthophyll cycle activity (Fig. 3). The PsbS/PSII core ratio was significantly reduced in *koLHCII* (−50%) compared to the wild type, while only slightly reduced in *koLhcb* mutants (−14% to 20%; Fig. 3A). The xanthophyll pool (VAZ) per Chls was higher in *koLHCII* (7.6 xanthophylls per 100 Chls) and *koLhcb* (9.4 xanthophylls per 100 Chls) compared to wild type (5.2 xanthophylls per 100 Chls) (Supplementary Table S3).

The de-epoxidation index (DI) kinetics, measured upon exposure of leaves to 1000 *µ*mol photons m^−2^ s^−1^ at RT, were similar in *koLHCII* and wild type (DI ∼0.3 upon illumination), but significantly faster in *koLhcb* leaves (DI ∼0.5; Fig. 3B). This is likely due to the much higher amount of Vio free in the lipid phase, which is rapidly converted into Zea under EL conditions (Dall’Osto et al. 2010). To estimate the light-driven proton motive force (pmf) across thylakoids, we measured the total amplitude of the electrochromic shift (ECS) signal (Cruz et al. 2004). The pmf was highest in wild type but significantly reduced in *koLHCII* (−20%) and *koLhcb* (−35%). However, all genotypes reached the half-saturation point of pmf at approximately 100 *μ*mol photons m^−2^ s^−1^ (Fig. 3C).

**Figure 4.**
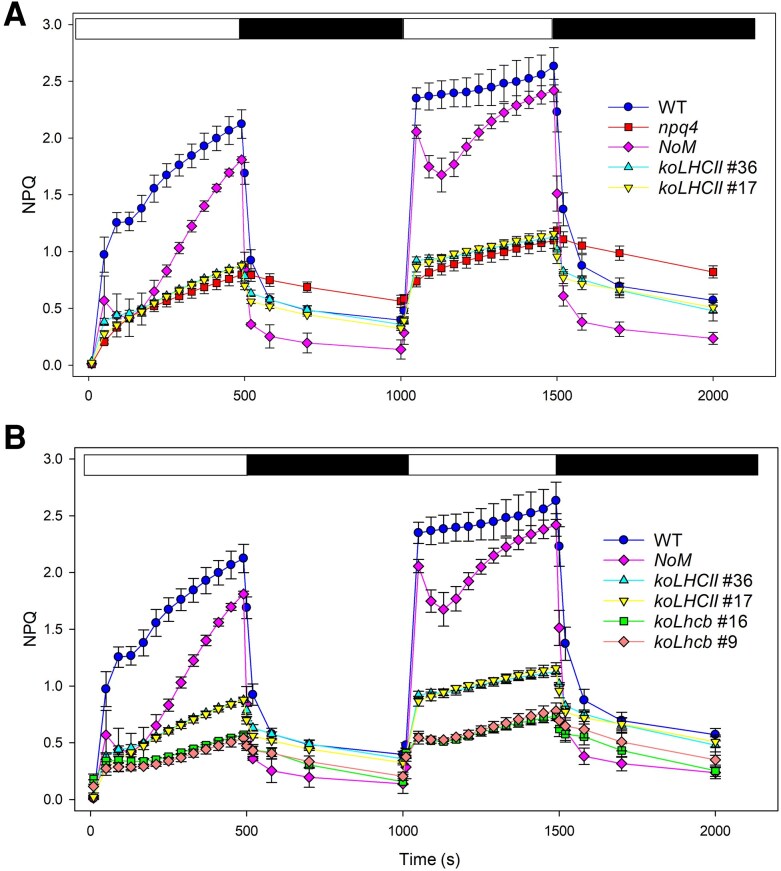
Kinetics of formation and relaxation of photoprotective energy dissipation in wild-type and *LHC* mutant leaves. NPQ kinetics were measured in selected genotypes during 2 consecutive illumination periods with white light (1,000 *μ*mol photons m^−2^ s^−1^) at RT. White and black bars represent light and dark periods, respectively. Data are expressed as mean ± Sd, *n* = 4 biologically independent plants. The experiments were repeated independently twice, yielding essentially identical results.

The NPQ activity of the different genotypes was investigated by measuring NPQ kinetics during exposure of dark-adapted leaves to EL (1000 *μ*mol photons m^−2^ s^−1^) for 8 min and following the fluorescence recovery in the dark (Fig. 4). Wild-type leaves exhibited a rapid NPQ phase that occurred within 1 min, which was dependent on the formation of trans-thylakoid ΔpH, followed by a slower phase linked to Zea formation (Hartel et al. 1996; Niyogi et al. 1998). Upon subsequent illumination, following a brief dark relaxation period, the slower phase was absent, consistent with high persistent Zea levels and slow re-epoxidation (Küster et al. 2023), which facilitated a faster NPQ rise during the second illumination (Andersson et al. 2001).

After both induction cycles, the *npq4* mutant exhibited the lowest qE and the slowest fluorescence recovery (qI). Deletions in PSII antenna system components resulted in varying impacts on NPQ. In *koLHCII* leaves, NPQ was significantly impaired, with a maximal qE amplitude of 0.8 compared to 2.2 in wild type, but no substantial increase in qI. Notably, a distinct fast relaxation component was observed in the dark for *koLHCII*, which was absent in *npq4* (Fig. 4). Since *npq4* lacks qE, this feature suggests that the monomeric Lhcbs in *koLHCII* mediate a qE component (Fig. 4A). In *koLhcb*, the NPQ rise was much slower than in *koLHCII* (Fig. 4B) and was similar to the quenching kinetics seen in *ch1 koLhcb5 npq4* leaves (Supplementary Fig. S6). In *ch1*, a rapidly reversible quenching activity was detected, significantly higher than in *koLHCII*. In contrast, the NPQ amplitude in *ch1 koLhcb5* was intermediate between the *ch1* and *koLhcb* lines (Supplementary Fig. S6), suggesting that monomeric Lhcb5 and/or other Lhcbs (Kim et al. 2009) are involved in catalyzing residual qE. It is important to note that Zea accumulated across all genotypes (Fig. 4B); therefore, the reduced NPQ amplitude is likely due to either altered ΔpH or the limited availability of Zea-binding sites.

Further genetic analysis was conducted to investigate the in vivo contribution of Zea, Lut, and PsbS to NPQ activity. *Arabidopsis koLHCII* mutants lacking Zea (*koLHCII npq1*), Lut (*koLHCII lut2*), or PsbS (*koLHCII npq4*) were generated using CRISPR-Cas9 genome editing. The mutations were confirmed through immunodetection and by HPLC analysis (Supplementary Fig. S7 and Tables S1 and S4).

**Figure 5.**
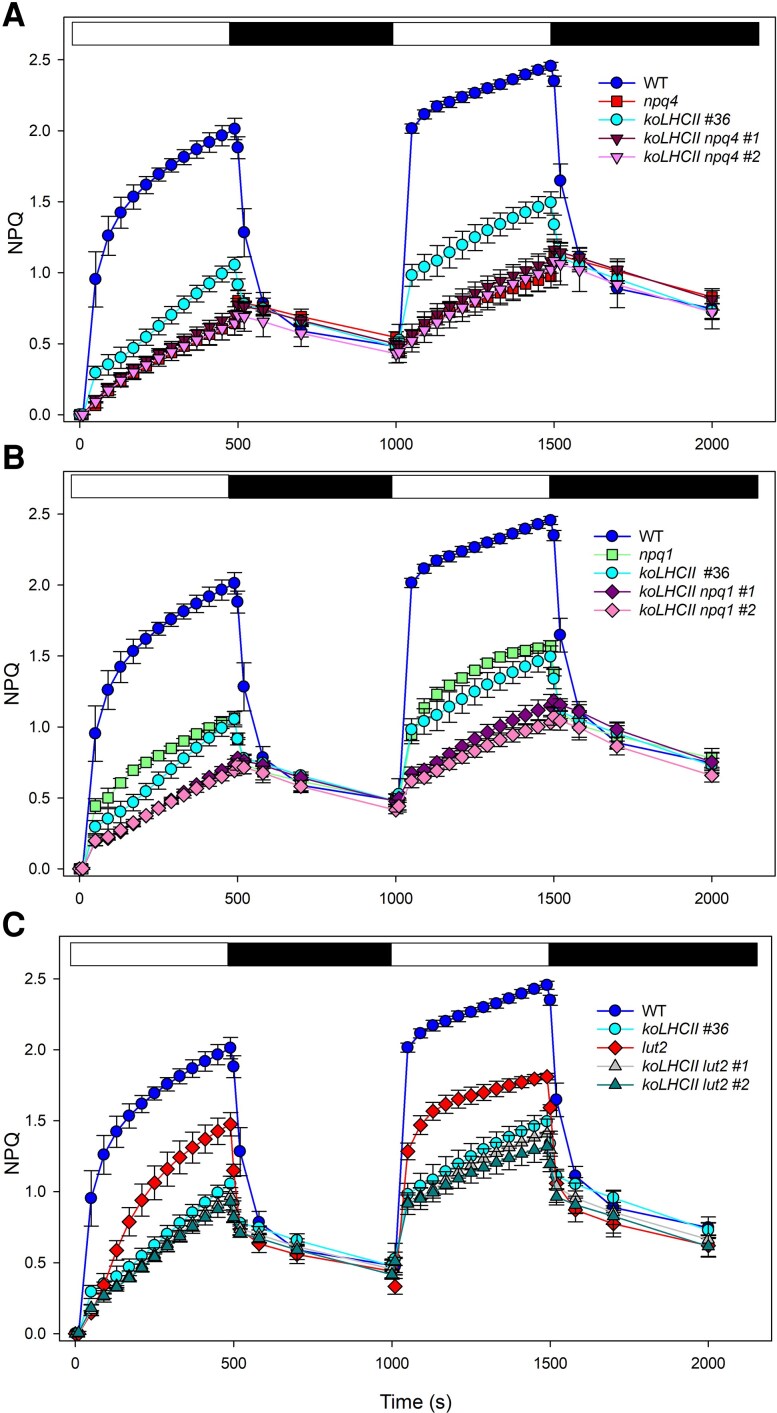
Kinetics of formation and relaxation of photoprotective energy dissipation in wild type and mutants *koLHCII npq* and *koLHCII lut2*. NPQ kinetics were measured in leaves during 2 consecutive periods of illumination with white light (1,000 *μ*mol photons m^−2^ s^−1^) at RT. White and black bars represent light and dark periods, respectively. Data are expressed as mean ± Sd, *n* = 4 biologically independent plants. The experiments were repeated independently twice, producing similar results.

Deleting PsbS resulted in a complete loss of NPQ activity in *koLHCII npq4* (Fig. 5A). The rapid phase of NPQ induction and relaxation was well-defined in *koLHCII npq1* and *koLHCII lut2* during both illumination periods (Fig. 5, B and C). In contrast, the slower phase, still present in *npq1* leaves, was significantly reduced in *koLHCII npq1* leaves, suggesting that Zea binding to residual monomeric Lhcbs modulates their quenching activity. However, in *lut2* leaves, the slower phase was equally reduced in both *koLHCII* and *koLHCII lut2* plants, indicating that the monomeric Lhcbs did not have Lut-binding sites active in NPQ. Overall, the double illumination experiment confirmed that NPQ in genotypes retaining only monomeric Lhcbs was entirely dependent on Zea and independent of Lut.

### Mapping the photoprotective response in plants lacking either monomeric or trimeric LHC

Previous results have shown that monomeric and trimeric Lhcb moieties contribute to the overall quenching activity in *Arabidopsis* leaves. However, assessing the contribution of each quenching site to NPQ is complex due to differences in the functional photon absorption cross-section of individual genotypes. To assess the specific roles of monomeric vs. trimeric Lhcbs in excess energy dissipation, we selected genotypes with similar levels of antenna proteins. Specifically, we focused on the *NoM koLhcb3* line, engineered to accumulate only the Lhcb1 + Lhcb2 complement. LHC content was quantified using Coomassie blue staining and densitometry, leading to the selection of 16 lines based on LHCII content, which ranged from 0.004 to 0.028 nmol LHC/µg Chl in thylakoids. Supplementary Figure S8 illustrates their NPQ activity.

**Figure 6.**
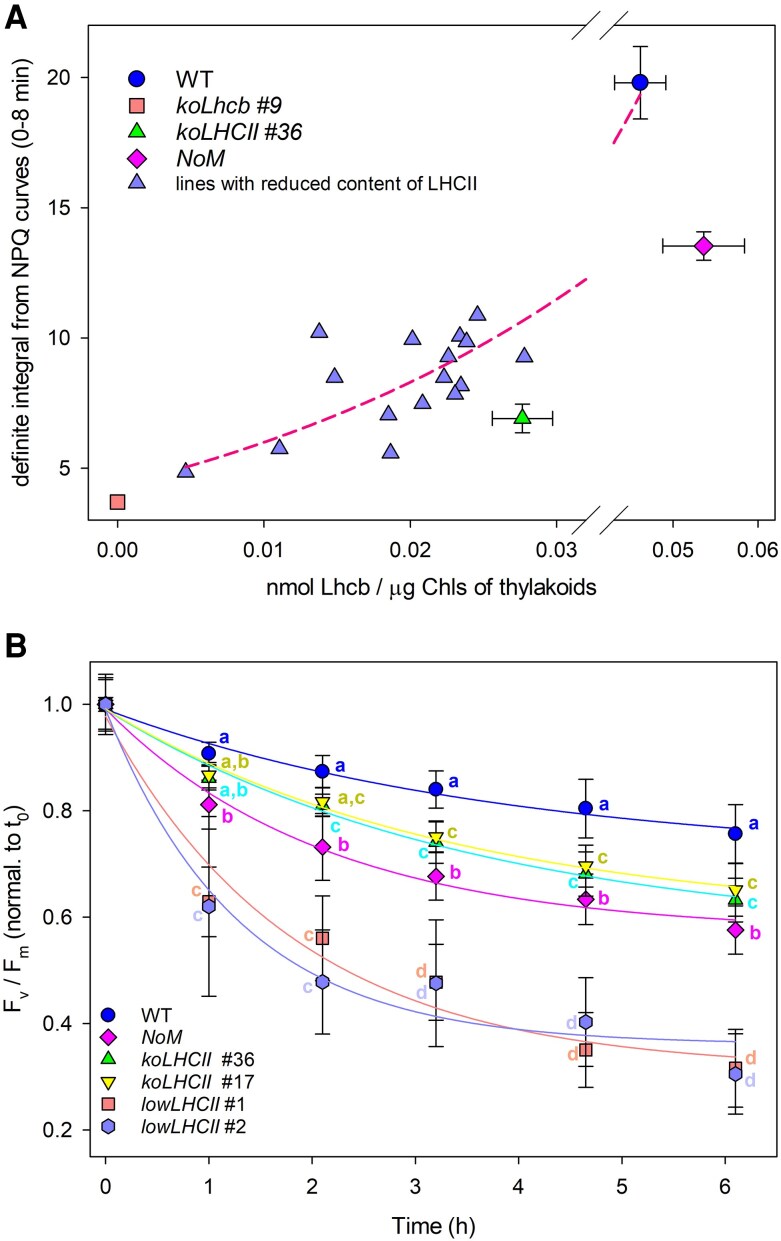
Correlation between photoprotective responses and LHC content in plants expressing either monomeric LHC or trimeric LHCII only. **A)** NPQ amplitude was measured in wild-type and mutant plants lacking specific LHC subunits (*NoM*, *koLHCII*, *koLhcb*), as well as in a population expressing varying level of Lhcb1-Lhcb2 complexes. For each plant, (i) LHC content was estimated via SDS-PAGE, Coomassie staining, and densitometric analysis, and (ii) definite integrals were calculated from NPQ curves within the range 0 to 8 min range (Supplementary Fig. S8B). Experimental points (magenta triangles) were analyzed for correlation analysis using a sigmoidal function. Data are expressed as mean ± Sd, *n* = 4 biologically independent plants. Each experimental point represents the LHCII content of the plant line and the corresponding definite integral from its NPQ trace. Leaves of *koLHCII* leaves, which express only monomeric LHC, showed significantly lower NPQ amplitude compared to leaves expressing a stoichiometric amount of LHCII (2-sided Student's *t*-test, *P* < 0.05). **B)** PSII photoinhibition (measured as F_v_/Fm decay) was monitored in wild-type and antenna-depleted mutant plants, exposed to 550 *µ*mol photons m^−2^ s^−1^, at 4 °C for 6 h. Data are expressed as mean ± Sd, *n* ≥ 12 biologically independent samples. Values marked with different letters significantly differ within the same time point (ANOVA followed by Tukey's post hoc test at a significance level of *P* < 0.05).

Figure 6A shows that NPQ activity in *koLhcb* steadily increased with LHCII accumulation. *koLHCII* lines exhibited significantly higher NPQ activity compared to *koLhcb* plants (Fig. 6A). However, when comparing the effect of LHCs accumulation on NPQ restoration, we observed that LHCII provided specifically higher NPQ activity than monomeric Lhcbs, particularly when considering the exact PSII core/LHC protein stoichiometry (Fig. 6A).

From lines engineered to accumulate a range of LHCII complement, we selected 2 *lowLHCII* lines, which contained one LHCII trimer per monomeric PSII core complex (Supplementary Fig. S9), thus had the same PSII/LHC ratio as *koLHCII*. First, we examined whether the expression of a subset of Lhcb influenced the organization of the photosynthetic apparatus (see Supplementary Fig. S10 for details). Results suggest that the mutations did not impact the remaining LHCs beyond their abundance, making these plants suitable candidates for investigating the role of LHCs in preventing photoinhibition. We therefore monitored the maximum photochemical yield of PSII (F_v_/F_m_) during 6 h of EL treatment at low temperature (550 *µ*mol photons m^−2^ s^−1^, 4 °C), a condition that exacerbates photooxidative stress (Shumbe et al. 2017) (Fig. 6B). Wild-type plants showed a gradual decrease in F_v_/F_m_ to 80% of the initial value (0.82). In contrast, *NoM* plants experienced a faster F_v_/F_m_ decline, while *koLHCII* was more resistant to photoinhibition (half-time of photoinhibition, t_1/2_: 1.4 h for *NoM* vs. 1.9 h for *koLHCII*). Interestingly, the *lowLHCII* lines with the same PSII/LHC ratio as *koLHCII* were more prone to photoinhibition (t_1/2_: 1.1 h). This suggests that the PSII phototolerance in *koLHCII* was significantly stronger than in the *lowLHCII* lines, despite the higher quenching capacity of LHCII compared to monomeric Lhcbs (Fig. 6A).

Since the F_v_/F_m_ ratio is highly specific for PSII activity, we conducted a more comprehensive assay by measuring Chl photobleaching kinetics in leaf discs exposed to intense light (1,800 *µ*mol photons m^−2^ s^−1^, 4 °C) for 14 h. The results (Supplementary Fig. S11) were consistent with those obtained from F_v_/F_m_ measurements in cold conditions.

## Discussion

Light-harvesting and excitation energy transfer regulation between PSII complexes in grana membranes are mediated by LHC proteins. These LHCII assemblies are modulated by xanthophyll cycle pigments and PsbS (Li et al. 2004) in response to lumenal pH. Absorbing excess energy can lead to photoinhibition, making the fine-tuning of excitation crucial for photoprotection (Bassi and Dall’Osto 2021). Identifying specific regulatory mechanisms within individual LHCs is challenging due to redundancy in the Lhcb subfamily (Jansson 1999). However, reverse genetics help dissect gene product properties. A particularly lively debate developed about the localization of quenching reactions, i.e. whether quenching occurs solely in LHCII or at multiple sites (Ruban and Horton 1992; Guardini et al. 2020; Nicol et al. 2021). This study analyzes mutant lines lacking specific Lhcb subgroups, incorporating mutations in qE-related genes involved in quenching regulation.

The analysis of multiple mutants reveals key findings:

First, excitation energy transfer between PSII in wild-type grana membranes exhibits cooperative behavior, essential to prevent excitons from re-emitting as fluorescence at closed RCs (Lavergne and Joliot 1996). This was thought to result from direct transfer between facing PSII core components within the supercomplex. However, in this study, despite all genotypes retained dimeric PSII, only the wild type and *koLHCII* showed strong cooperativity. In contrast, *koLhcb* and *NoM* did not (Fig. 2 and Table 2), indicating that monomeric Lhcbs are crucial for cooperativity.

Second, monomeric Lhcbs play a significant role in NPQ. The *koLhcb*, lacking both trimeric LHCII and monomeric Lhcbs, exhibited NPQ activity at only 22% of wild-type values (Fig. 4). Despite comparable PsbS, Zea content, and pmf amplitude between *koLHCII* and *koLhcb* plants (Fig. 3), the differences in NPQ suggest that quenching site(s) within the monomeric Lhcbs, including the Chl cluster *a*603-*a*609-*a*616 in Lhcb4 (Guardini et al. 2020), have a significant activity. The results from Townsend et al. (2018) on *NoM* plants indicate that full qE can be attained even in the absence of minor antenna complexes, suggesting that the remaining LHCs may compensate for their absence. Trimeric LHCII also hosts PsbS-dependent quenching sites, as evidenced by a ∼68% reduction in NPQ activity in *koLHCII* compared to wild type (Fig. 4). This reduction may be slightly overestimated due to somehow lower PsbS levels and pmf amplitude (Fig. 3). Notably, the reduced LHCII complement may require less PsbS for saturation at PsbS-LHCII binding sites, as PsbS is sub-stoichiometric relative to PSII RC (McKenzie et al. 2020). This suggests PsbS may trigger quenching reactions across multiple supercomplexes (Bennett et al. 2018).

Third, Lhcb5 contributes to NPQ in *ch1* plants, as evidenced by the substantial reduction in NPQ activity observed in *ch1 koLhcb5* vs. *ch1* (Supplementary Fig. S6). These genotypes also exhibited significant differences in biomass accumulation (Supplementary Fig. S1), suggesting that Lhcb5, which remains stable without Chl *b* (Havaux et al. 2004), was excitonically connected to PSII RC. Interestingly, de Bianchi et al. (2008) found no NPQ reduction activity in *koLhcb5* compared to wild type, implying that Lhcb5 may not interact with PsbS in wild type, possibly due to shielding by LHCII components in the PSII supercomplex, which is lacking in *ch1*. Alternatively, the different role of Lhcb5 in the wild type compared to the *ch1* genetic background may be attributed to this antenna's ability to form parallel-associated PSII arrays (Vánská et al. 2025). Current evidence, along with previous findings (Kim et al. 2009), suggest that Chl *b*-deficient mutant *ch1* should not be used as a reliable proxy for LHC-less plants.

Fourth, Zea activates quenching responses in both PSII antenna domains. Pre-loading thylakoids with Zea enhanced the rapid phase of quenching in *koLHCII*, although this effect was significantly diminished in *koLHCII npq1* plants (Fig. 5B), suggesting that Zea binding to monomeric Lhcbs modulated their quenching activity. In *NoM* plants, quenching is fully Zea-dependent (Dall’Osto et al. 2017), indicating that Zea catalyzes quenching in both monomeric and trimeric LHC domains. Zea triggers a conformational change in Lhcb4, promoting quenching by xanthophyll ligand at site L2, as shown by in vivo site-directed mutagenesis (Guardini et al. 2020). Consistently, Zea binding at the L2 site of Lhcb4 reduces the fluorescence lifetime of the complexes and decreases ^3^Car* formation (Sardar et al. 2022), emphasizing Zea crucial role in photoprotection through its interaction with LHCs. Among monomeric antennae, Lhcb5 and Lhcb6 were the most efficient in xanthophyll exchange in vivo (Morosinotto et al. 2002). More recent findings (Xu et al. 2015) suggest that Zea does not bind to the internal sites of the antennas, but instead acts in between the complexes.

Notably, NPQ activity was retained to varying degrees whenever PsbS and Zea were present, even in *koLHCII* and *koLhcb* genotypes lacking LHCII (Fig. 4). The interaction between PsbS and monomeric Lhcbs likely induces conformational change(s) promoted by Vio/Zea exchange, facilitating interactions between Chl *a* ligands and xanthophyll at binding site L2 (Ahn et al. 2008). Additionally, PsbS interact with LHCII (Nicol and Croce 2021), as demonstrated by the absence of qE in the *NoM npq4* mutant (Dall’Osto et al. 2017). This interaction may depend on Zea, potentially at the interface between PsbS and its LHCII interactor (Wilk et al. 2013).

Topological analysis suggests that NPQ induction also involves the dissociation of the C_2_S_2_M_2_ supercomplex (Betterle et al. 2009; Johnson and Ruban 2010). However, robust quenching observed in *NoM* (Fig. 4), which lacks PSII supercomplexes, and in *lowLHCII* plants (Supplementary Fig. S9) confirms that LHCII trimers serve as quenching sites independent of their assembly in PSII supercomplexes (Figs. 1B and 4).

PsbS-dependent quenching in mutants with subsets of the PSII antenna system, whether monomeric or trimeric LHCs, requires Zea for full activity, but is unaffected by the *lut2* mutation (Fig. 5) (Dall’Osto et al. 2017). This finding is unexpected, as previous studies suggested qE dependence on Lut in *Arabidopsis npq1 lut2* genotypes (Niyogi et al. 2001) and enhancement in the *szl1* mutant (Li et al. 2009). Regarding trimeric LHCII, Vio and Lut can substitute for each other at the same quenching site(s), as indicated by the identical NPQ competence in *NoM lut2* and *NoM* genotypes (Supplementary Fig. S12). Since *NoM* and *NoM lut2* differ in the occupancy of LHCII xanthophyll binding sites L1 and L2 by Lut vs. Vio, respectively, but exhibited the same Zea-dependent quenching activity, we conclude that Zea-dependent quenching is driven by Zea binding at site V1, the only site undergoing Vio→Zea exchange under EL (Caffarri et al. 2001). Zea has been shown to promote LHCII aggregation, with the degree of aggregation correlating with the extent of NPQ (Shukla et al. 2020). The process of LHCII-Zea protonation and aggregation is proposed to trigger changes in thylakoid membrane thickness that occur during NPQ (Johnson et al. 2011a).

The loss of Lut dependence on NPQ activity in both *NoM* and *koLHCII* is noteworthy. The *lut2* mutation causes monomerization of trimeric LHCII and destabilizes PSII supercomplexes (Lokstein et al. 2002). This suggests that the reduced qE in the *lut2* plants may result from PsbS-LHCII interactions inducing quenching in monomers rather than trimers. Alternatively, the decreased quenching could arise from altered PSII-LHCII segregation in *lut2* in response to EL, rather than from impaired quenching reaction(s) within Lhcb. Nevertheless, the identity of the xanthophyll at site L1 did not determine NPQ activity, as shown by unaltered qE in *koLHCII lut2* (Fig. 5C) and *NoM lut2* plants (Dall’Osto et al. 2017). While Lut has been suggested to function as a quencher in monomeric and trimeric LHCs (Johnson et al. 2011b) during aggregation in vitro (Ruban et al. 2007), the unchanged quenching activity in *koLHCII lut2* implies that at least one qE component related to LHCII is independent of clustering and aggregation in vivo.

### Role of distinct Lhcbs in excitation energy transfer and trapping

Following normalization to the same cross-section (Fig. 2C), Chl fluorescence rise was strictly identical in *koLHCII* and wild type, consistent with the connectivity parameter *J*, which reflects the long-range organization of antenna pigments connecting PSII RCs (Joliot and Joliot 2003). High connectivity allows energy transfer between multiple LHCs, enabling excitons to transfer to open RCs rather than being emitted as fluorescence (Lavergne and Trissl 1995). *J* values were high (1.75 to 2.05) in wild-type and *koLHCII* plants, but significantly lower in *NoM* (0.79) and *koLhcb* (0.60), indicating decreased connectivity (Table 2).

Unexpectedly, the F_v_/F_m_ ratio of *koLHCII* (0.76, Table 2) was lower than wild type (0.82). Based on the lake model (Genty et al. 1989), this suggest that the reduced number of pigments visited before trapping by RCs should correspond to a lower F_0_/F_v_ ratio, which contradicts experimental values. This discrepancy may arise from a higher F_0_ in the *koLHCII*, due to increased PSI Chl contribution as the PSII outer pigment pool shrinks and/or to the presence of unconnected pigments.

In *NoM*, the optical cross-section measured with DCMU was 1.9 times larger than wild type (Fig. 2B and Table 2). However, in the absence of DCMU, the fluorescence rise, which mainly reflects the rate of reduction of the PQ pool, slowed down significantly (by 3.2-fold). As the cross-section in the *NoM* is 1.9 times larger (Table 2), this suggests that the ratio of soluble PQ molecules to active PSII RC is roughly 1.9 × 3∼6 times larger in *NoM* than in wild type. Ultrastructure analysis on *NoM* revealed a heterogeneous distribution of antenna and RCs (Dall’Osto et al. 2020), with some RC dimers included in large patches devoid of trimers, and others randomly distributed in the membrane in close contact with trimers, which are present in excess with respect to the wild type. Such a heterogeneous spatial distribution could lead to varying RC cross-section, while the absence of a slow phase in fluorescence rise in the presence of DCMU suggests that patches of dimers may contain inactive RCs.

The cross-section in *koLhcb* was 4 times lower than in wild type (Table 2) due to the loss of peripheral antennae. *NoM* exhibited similar slowing of fluorescence rise kinetics, which reflects comparable ratios of PQ pool to RCs in both strains.

Fluorescence kinetics in *koLhcb* demonstrated low connectivity, contrasting previous reported (Diner and Wollman 1979) of similar connectivity in a cyanobacterial strain lacking accessory pigments and in *Chlorella*. It is possible that the excitation energy in PSII core complexes in *koLhcbs* is quenched due to spill-over to PSI, possibly due to changes in the lateral heterogeneity between PSI and PSII domains.

Unexpectedly, Chl fluorescence decays in wild type and *koLHCII* were identical in the absence of DCMU, after the saturating light pulse (Fig. 2D). The fact that these genotypes differ in many respects while retaining a dimeric organization of the PSII core complex suggests that the minimum unit determining the PQ diffusion range is the PSII core dimer itself.

### Feedback de-excitation vs. phototolerance

Quenching reactions reduce excess energy accumulation in PSII, thereby providing photoprotection. This raises the question of whether the location of quenching sites within the antenna system affects PSII quantum yield under EL. *NoM* plants exhibited a faster rate of PSII photoinhibition compared to wild type (Fig. 6B), indicating that monomeric Lhcbs enhance phototolerance and that an over-accumulation of trimeric LHCII cannot substitute for their function. Increased sensitivity in *NoM* to photooxidation was associated with elevated ^1^O_2_ release (Dall’Osto et al. 2020) due to excess ^1^Chl* in poorly connected LHCIIs (Dall’Osto et al. 2014). In contrast, xanthophylls in LHCs enhance ^1^O_2_ scavenging (Johnson et al. 2007), making LHC-rich thylakoids more resilient to EL.

To elucidate the relative importance of ROS scavenging vs. other protective mechanisms, we created the *lowLHCII* genotype which, like *NoM*, lacks monomeric Lhcb but matches *koLHCII* in PSII/LHC stoichiometry. The order of photosensitivity, i.e. *lowLHCII* >> *NoM* > *koLHCII* > WT, confirming that monomeric Lhcbs are more effective at preserving PSII from photoinactivation than trimeric LHCII. Photoprotective mechanisms proposed for PSII include (i) thermal dissipation of ^1^Chl* via qE, (ii) ROS scavenging, and (iii) quenching of ^3^Chl* (Niyogi 1999). The higher NPQ in *lowLHCII* vs. *koLHCII* (Supplementary Fig. S9) suggests that qE is not the main factor behind increased phototolerance; instead, monomeric Lhcbs may shield the PSII core from ROS or peroxy-lipid radicals released in peripheral LHCII or PSI. However, purified monomeric antennae are more susceptible to photooxidation than LHCII (Dall’Osto et al. 2006). The higher efficiency of ^3^Chl* → ^3^Car* energy transfer in trimeric LHCII (Mozzo et al. 2008) supports the notion that monomeric antennae might control ^3^Chl* formation, enhancing PSII resistance to EL. Beyond β-carotene, which plays a crucial role in protecting P680 (Krieger-Liszkay et al. 2008), xanthophylls bound to monomeric Lhcbs may promote phototolerance, by dissipating excitation energy from the RC or facilitating ^3^Chl* energy transfer from the RC (Nayak et al. 2002). In conclusion, monomeric antennae facilitate qE and contribute to maintaining PSII quantum yield under photooxidative conditions. This explains the greater photosensitivity in plants, e.g. *NoM*, where LHCII replaces minor antennae and confirms the unique function of monomeric Lhcbs beyond qE in mitigating photoinhibition. We propose this mechanism relies on Zea, likely through its binding to monomeric complexes, modulating ^3^Chl* yield in vivo and preventing ROS release.

## Materials and methods

### Plant material and growth conditions

Wild-type plants *A. thaliana* (Col-0) and mutant lines *NoM*, *koLhcb3*, *ch1*, *ch1 koLhcb5*, and *npq4* were obtained as previously described (Li et al. 2000; Havaux et al. 2007; Dall’Osto et al. 2017). *NoM koLhcb3* and *ch1 koLhcb5 npq4* were obtained by crossing single mutants and selecting progeny by immunoblotting. *koLHCII*, *koLhcb*, *koLHCII npq4*, *koLHCII npq1*, and *koLHCII lut2* lines were obtained by genome editing as reported in Ordon et al. (2017, 2020). Plants were transformed with *Agrobacterium tumefaciens* (strain GV3101). Seedlings were tested for resistance to applications of the antibiotic hygromycin (25 mg l^−1^). For each genotype, independent transformants (T1 generation) were self-fertilized, and absences of proteins were confirmed in the T3 generation by immunotitration. *LowLHCII* lines were derived from crosses between *NoM koLhcb3 koLhcb1* (Ordon et al. 2020) and *NoM koLhcb3 koLhcb2* (Guardini et al. 2022), and the T2 segregant population was analyzed based on Lhcb1 and Lhcb2 levels. Plants were grown in a phytotron for 6 weeks under the following conditions: 150 *μ*mol photons m^−2^ s^−1^, 23 °C, 70% humidity, and a day/night cycle of 8/16 h. Supplementary Table S5 summarizes the key characteristics of the mutant lines analyzed.

### Membrane isolation

Stacked thylakoid membranes were isolated as previously described (Casazza et al. 2001). Grana-enriched membranes have been isolated from dark-adapted samples (Morosinotto et al. 2010).

### Pigment analysis

To measure zeaxanthin accumulation, detached leaves were exposed to 1000 *μ*mol photons m^−2^ s^−1^ at room temperature (RT, 22 °C). Pigments were extracted using 85% acetone buffered with Na_2_CO_3_ and quantified either by HPLC (Jasco Extrema LC-4000) as described by Gilmore and Yamamoto (1991) or through the deconvolution of spectra from acetone extracts (Croce et al. 2002).

### Spectroscopy

Absorption measurements were conducted at RT using an SLM Aminco DW-2000 spectrophotometer. The light-induced pmf and ECS_t_ (ECS) were estimated from changes in leaf absorbance at 520 nm, using a JTS10 (Biologic Science Instruments), following the method of Livingston et al. (2010).

### Gel electrophoresis and immunoblotting

SDS-PAGE was carried out using either the Tris-Tricine buffer system (Schägger and von Jagow 1987) or a modified Laemmli system (Ballottari et al. 2004). Non-denaturing Deriphat-PAGE was performed as in Havaux et al. (2004) and BN-PAGE and 2D electrophoresis as in (Järvi et al. 2011). For immunotitration (Towbin et al. 1979), proteins were electroblotted onto nitrocellulose membranes and detected using an alkaline phosphatase-conjugated antibody (Sigma-Aldrich A3687). The primary antibodies used were α-PsbB/CP47 (AS04 038), α-Lhcb1 (AS01 004), α-Lhcb2 (AS01 003), α-Lhcb3 (AS01 002), α-ATPase (AS05 085), α-cytochrome *f* (AS20 4377), and α-PsaA (AS06 172) from Agrisera and α-PsbS, α-PsbP, α-Cyt *f,* α-Lhcb4, α-Lhcb5, α-Lhcb6, and α-VDE, which were custom-made.

### Analysis of Chl fluorescence

Chl fluorescence parameters were measured in leaves at RT using a Dual-PAM (Heinz-Walz) and calculated according to (Kramer et al. 2004; Baker 2008). Fluorescence kinetics were also measured in vacuum-infiltrated leaves with 3·10^−5^ M 3-(3,4-dichlorophenyl)-1,1-dimethylurea (DCMU), 10 mm HEPES pH 7.5 and 100 mm sorbitol. The two-thirds time of the fluorescence rise was used to measure the functional antenna size of PSII (Malkin et al. 1981). The fluorescence induction in DCMU-treated leaves was fitted by the function F(f, F_v_, I, Φ_PSII_, *J*) based on a sigmoidal fluorescence induction model (Lavergne and Trissl 1995), where *J* is the connectivity parameter that determines the shape of the curve. Fast light-induced Chl fluorescence kinetics, reported in Fig. 2, were measured on intact leaves at RT with a Joliot-type spectrofluorometer (JTS10; Biologic).

### Determination of sensitivity to photooxidative stress

Photooxidative stress was induced in detached leaves exposed to EL (550 *μ*mol photons m^−2^ s^−1^, 4 °C) using a 150 W halogen lamp. The decay kinetics of the maximal quantum yield of PSII photochemistry (F_v_/F_M_) were monitored (Havaux et al. 2004). In vivo photobleaching was induced in leaf discs exposed to 1,800 *μ*mol photons m^−2^ s^−1^, 4 °C (Dall’Osto et al. 2012).

### Electron microscopy and image analysis

Leaf fragments from the tip part of fully developed leaves were fixed in 3% glutaraldehyde in 0.1 m cacodylate buffer pH 6.9. Transmission electron microscopy analysis was conducted using a FEI Tecnai T12 electron microscope operating at 100 kV accelerating voltage. EM images were analyzed with *ImageJ* software (Schneider et al. 2012).

### Sucrose gradient fractionation

Sucrose gradient ultracentrifugation was conducted as described in Dall’Osto et al. (2006).

### Statistics

Statistical significance was determined using either Student's *t-test* or ANOVA in GraphPad Prism (see the figure legends for details).

### Accession numbers

Sequence data from this article can be found in the *Arabidopsis* Genome Initiative or GenBank/EMBL databases under accession numbers At1g29920 (*Lhcb1.1*), At1g29910 (*Lhcb1.2*), At1g29930 (*Lhcb1.3*), At2g34430 (*Lhcb1.4*), At2g34420 (*Lhcb1.5*), At2g05100 (*Lhcb2.1*), At2g05070 (*Lhcb2.2*), At3g27690 (*Lhcb2.3*), At5g54270 (*Lhcb3*), At5g01530 (*Lhcb4.1*), At3g08940 (*Lhcb4.2*), At4g10340 (*Lhcb5*), At1g44446 (*cao*), At1g08550 (*npq1*), At1g44575 (*npq4*), and At5g57030 (*lut2*). The KO lines used in the work were obtained from the NASC under the stock numbers N376476 (*koLhcb4.1*), N877954 (*koLhcb4.2*), N514869 (*koLhcb5*), N520342 (*koLhcb3*), and N524295 (*ch1*). K.K. Niyogi kindly provided *npq4* mutant.

## Supplementary Material

kiaf588_Supplementary_Data

## Data Availability

All data supporting the findings of this study are included in the article and its supplementery materials. Additional data or information can be obtained from the corresponding authors upon reasonable request.

## Supplementary data

The following materials are available in the online version of this article.

**Supplementary Figure S1.** Phenotype of wild-type and mutant plants.

**Supplementary Figure S2.** Immunoblotting was used to quantify the major photosynthetic subunits in both wild-type and mutant thylakoids.

**Supplementary Figure S3.** Analysis of pigment-binding complexes separated by non-denaturing Deriphat-PAGE.

**Supplementary Figure S4.** Biochemical characterization of wild type and *koLHCII* mutant plants.

**Supplementary Figure S5.** Analysis of Chl fluorescence and energy partitioning during photosynthesis in wild-type and mutant leaves at RT.

**Supplementary Figure S6.** Kinetics of formation and relaxation of photoprotective energy dissipation in wild-type, *ch1*, and *LHC* mutant leaves.

**Supplementary Figure S7.** Characterization of *koLHCII npq1* and *lut2* lines.

**Supplementary Figure S8.** Characterization of *NoM* lines expressing varying amounts of the LHCII antenna.

**Supplementary Figure S9.** Photoprotective energy dissipation of *lowLHCII* lines.

**Supplementary Figure S10.** Characterization of *koLHCII*, *lowLHCII* and *NoM* mutant plants.

**Supplementary Figure S11.** Photoprotective role of LHC.

**Supplementary Figure S12.** Kinetics of formation and relaxation of photoprotective energy dissipation in wild-type and mutants *NoM*, *koLHCII*, and *lut2*.

**Supplementary Table S1.** Target sites used for sgRNA design.

**Supplementary Table S2.** F_v_/F_m_ values determined in leaves from wild type and mutant plants.

**Supplementary Table S3.** Total VAZ (violaxanthin + antheraxanthin + zeaxanthin) content in leaves from wild-type and mutant lines.

**Supplementary Table S4.** Pigment content determination for wild type, *koLHCII*, *npq1*, and *lut2* lines.

**Supplementary Table S5.** Table summarizing the *Arabidopsis* mutants used in this study.

## Dive Curated Terms

The following phenotypic, genotypic, and functional terms are of significance to the work described in this paper:

LUT2 Gramene: AT5G57030
LUT2 Araport: AT5G57030

NPQ1 Gramene: AT1G08550
NPQ1 Araport: AT1G08550

CAO Gramene: AT2G47450
CAO Araport: AT2G47450

LHCB5 Gramene: At4g10340
LHCB5 Araport: At4g10340

electron CHEBI: CHEBI:10545

CH1 Gramene: AT1G44446
CH1 Araport: AT1G44446

npq4 Gramene: AT1G44575
npq4 Araport: AT1G44575

LHCB3 Gramene: AT5G54270
LHCB3 Araport: AT5G54270

DCMU CHEBI: CHEBI:116509

SZL1 Gramene: AT3G10230
SZL1 Araport: AT3G10230
