# Supplementary material for: Mapping light-harvesting and photoprotection responses in the Photosystem II antenna system of higher plants
Source: Plant Physiol. 2025 Nov 13;199(4):kiaf588. doi: 10.1093/plphys/kiaf588 (PMC12671495; doi:10.1093/plphys/kiaf588)
Supplement: kiaf588_Supplementary_Data [file kiaf588_supplementary_data.pdf]

Supplementary Data

Supplementary Figure S1

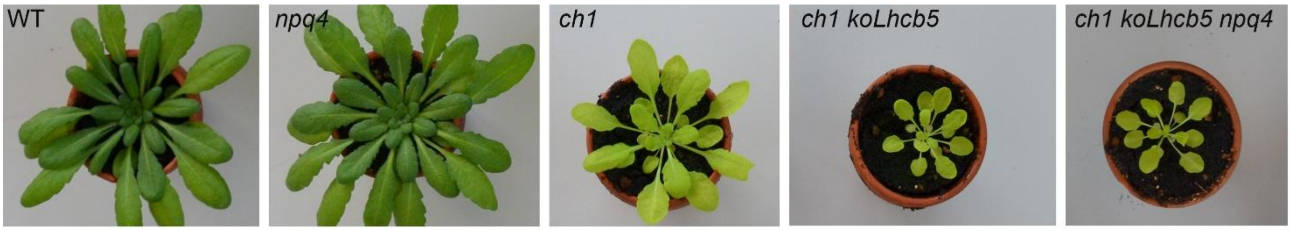

	Chl *a* / *b*	Chl / Car	µg Chl cm^{-2}	Fresh weight (g)
**WT**	3.51 ± 0.09^a	3.69 ± 0.21^a	19.4 ± 0.5^a	2.03 ± 0.19^a
npq4	3.40 ± 0.02^a	3.66 ± 0.13^a	21.1 ± 1.4^a	2.28 ± 0.46^a
ch1	-	2.77 ± 0.02^b	6.55 ± 0.54^d	0.30 ± 0.06^c
ch1 koLhcb5	-	2.57 ± 0.02^b	4.50 ± 0.23^e	0.07 ± 0.02^d
ch1 koLhcb5 npq4	-	2.59 ± 0.08^b	3.87 ± 0.27^e	0.07 ± 0.02^d

Supplementary Figure S1. Phenotype of wild type and mutant plants. (*upper panel*) Plants were grown for 6 weeks under conditions of 150 µmol photons m^{-2} s^{-1}, 23/19 °C (day/night), with an 8/16-hour light/dark cycle. (*lower panel*) Pigment content of leaves from *Arabidopsis* WT, *npq4* and *ch1* mutant lines. The Chl/Car represents the molar ratio between chlorophylls (*a* + *b*) and carotenoids. Fresh weight refers to plants grown for 6 weeks under control conditions. All data are expressed as mean ± s.d., *n* = 5 biologically independent plants. Values marked with different letters significantly differ within the column (ANOVA, followed by Tukey's post-hoc test at a significance level of $P < 0.05$). The experiments were independently repeated twice, yielding similar results. WT image in this Figure is the same as that in Figure 1A

Supplementary Figure S2

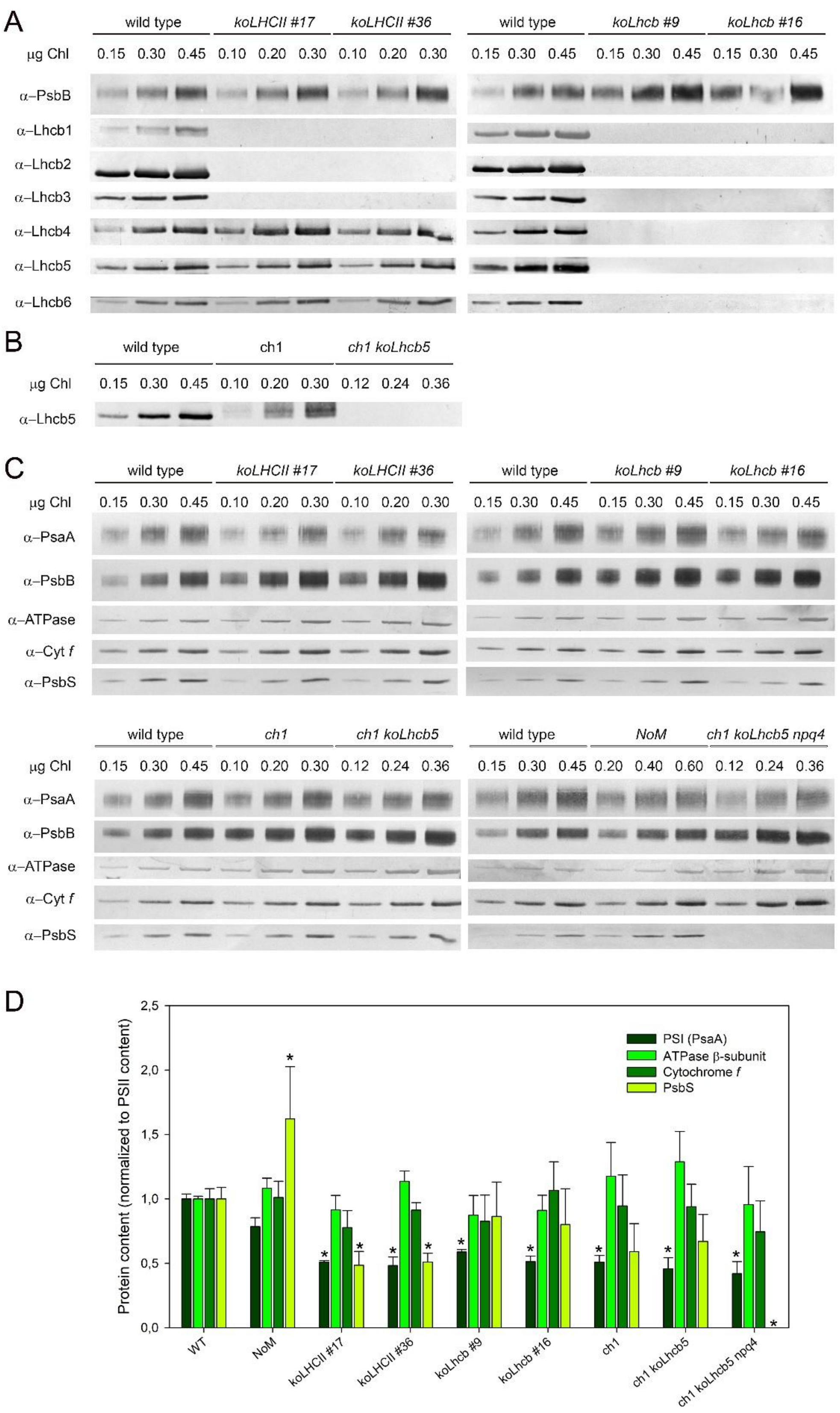

Supplementary Figure S2. Immunoblotting was used to quantify the major photosynthetic subunits in both wild type and mutant thylakoids. The analysis employed antibodies specific to individual gene products: Lhcb proteins and the PSII core subunit PsbB (CP47) (panels A, B), as

well as subunits of the primary thylakoid supercomplexes (PsaA, PsbB, cytochrome *f*, ATPase β subunit, panel C). (D) The data for each subunit were normalized to PSII core levels (PsbB content) and expressed relative to wild type values. The amount of Chl loaded in each lane is indicated. Comparisons between wild type and mutant samples were made only when both were loaded on the same SDS–PAGE slab gel. All data are expressed as mean ± s.d., $n = 3$. Asterisks (*) mark values that significantly differ from wild type (ANOVA followed by Tukey's post-hoc test at a significance level of $P < 0.05$).

Supplementary Figure S3

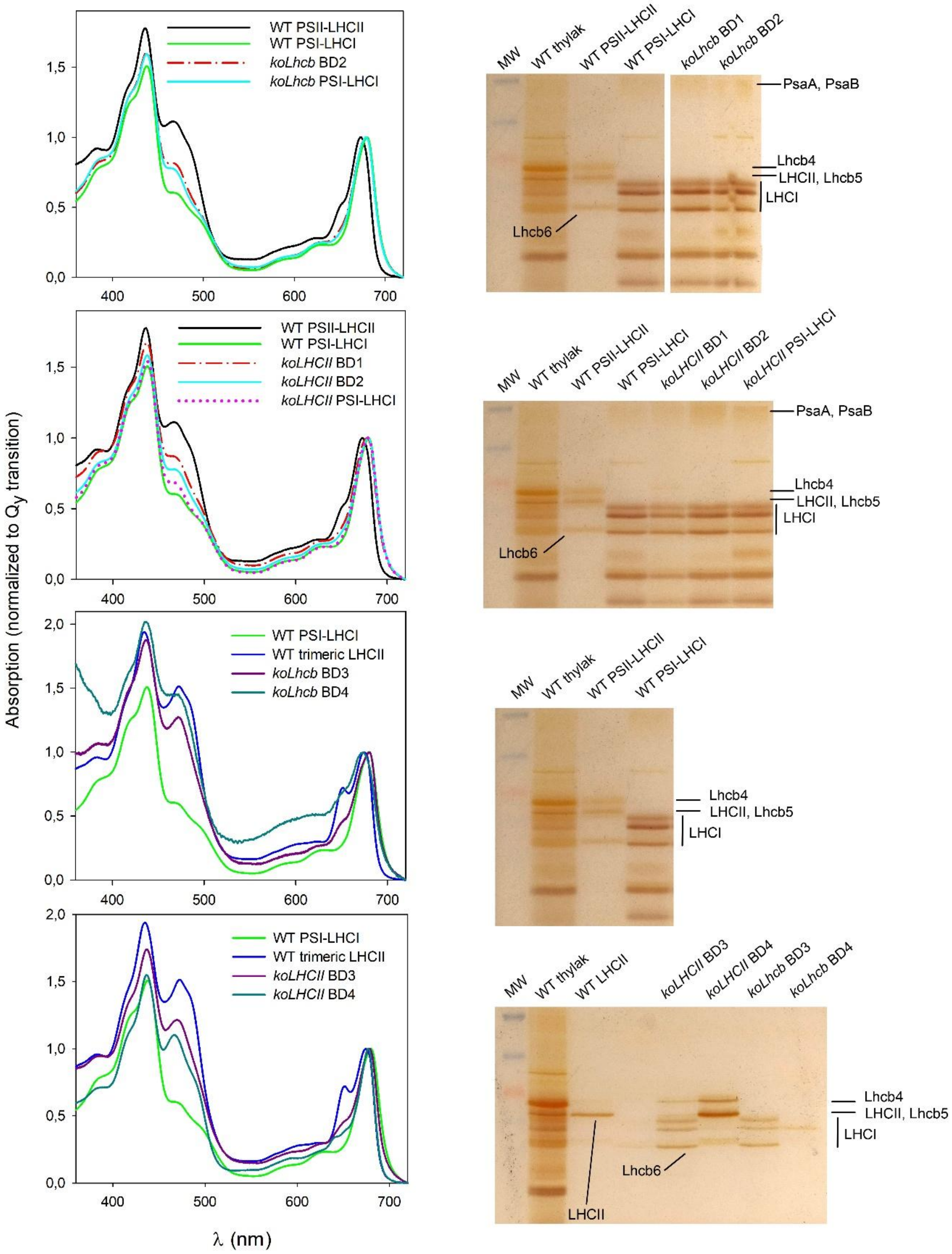

Supplementary Figure S3. Analysis of pigment-binding complexes separated by non-denaturing Deriphat-PAGE (refer to Figure 1B). Chl-binding complexes were extracted from the acrylamide matrix and further characterized by absorption spectroscopy at RT (*left column*). Additionally, the protein composition of specific bands was analyzed using SDS-PAGE and silver

staining (*right column*). Fractions BD1 and BD2 contained PSI-LHCI supercomplexes with increasing LHC content, as indicated by (i) the red shift of the Q_y transition, and (ii) a lower Chl *a/b* ratio compared to PSI-LHCI supercomplex from WT thylakoids. Major subunits identified in all these bands were PsaA/PsaB (PSI core complex) and LHCI (PSI antennae). BD3 and BD4 from *koLhcb* contained LHCI, likely in a dimeric form in BD3 (exhibiting electrophoretic mobility between trimeric LHCII and monomeric Lhcb), and monomeric in BD4. BD3 from *koLHCII* was enriched in LHCI subunits, along with dimeric states of the Lhcb complex (Lhcb4/Lhcb5). In BD4 from *koLHCII*, the proportion of monomeric LHCI was lower than in BD3, while monomeric Lhcb4, Lhcb5 and Lhcb6 were predominant. It is important to note that in the PAGE buffer system used, Lhcb1/2 and Lhcb5 co-migrate. Therefore, BD4 from *koLHCII* is considered to contain only Lhcb5 (Figures S2 and S4 confirm the absence of LHCII components in this genotype). The same 'WT thylak,' 'WT PSII-LHCII,' and 'WT PSI-LHCI' lanes were used as references for electrophoretic separation patterns in all three of the initial panels.

Supplementary Figure S4

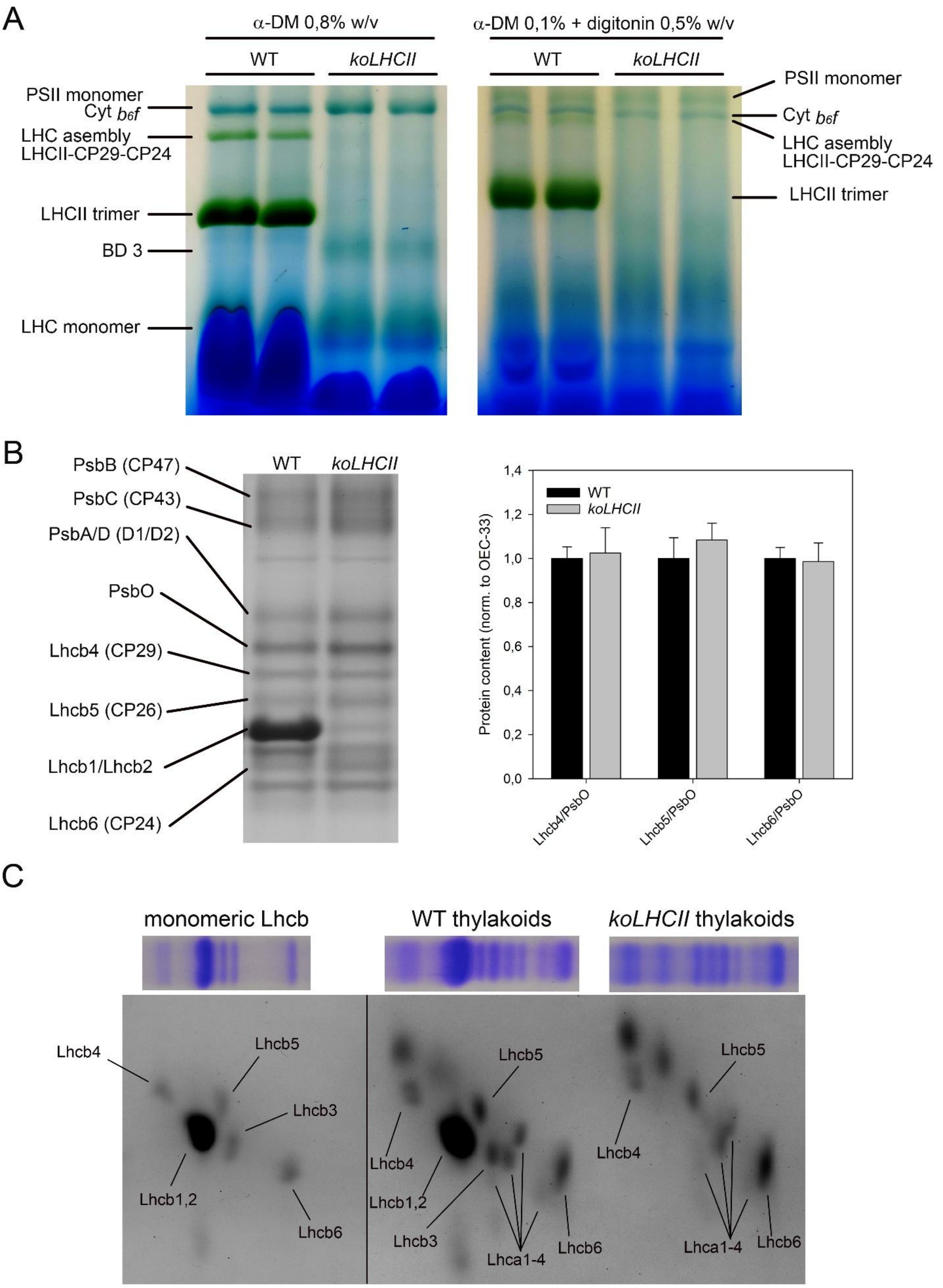

Supplementary Figure S4. Biochemical characterization of wild type and *koLHCII* mutant plants. (A) Pigment–protein complexes were separated by blue-native PAGE after solubilizing thylakoids with either 0.8% w/v α-DM (dodecyl-D-maltopyranoside) or 0.1% w/v α-DM + 0.5% w/v digitonin. Selected photosynthetic complexes are labeled. (B) (*left panel*) SDS–PAGE analysis of thylakoid proteins was performed using the Tris-Tricine buffer system and Coomassie staining. Note that the faint band migrating alongside Lhcb1/2 in *koLHCII* thylakoids, exhibited different electrophoretic mobility when analyzed with an alternative PAGE buffer system (see panel C). (*right panel*). Densitometric analysis showing the relative abundance of Lhcb4/Lhcb5/Lhcb6 in thylakoids. The data for antenna subunits were normalized to the core complex content (PsbO, 33 kDa subunit of the OEC), and values expressed as a percentage of the corresponding WT content. All data are expressed as mean ± s.d., $n = 4$; statistical analysis (Student's *t* test, $P < 0.05$) revealed no significant differences between WT and mutant. (C) 2D SDS–PAGE separation of the Lhcb region of thylakoids, allowing investigation of the depletion of specific Lhcb subunits in mutant plants**.** A mixture of monomeric Lhcb complexes were included as references. The PAGE buffer system used were: for the 1st dimension, a modified Laemmli system (Laemmli 1970); and for the 2nd dimension, Tris-Tricine buffer system (Schägger and von Jagow 1987) (see methods for details). The identity of each spot was attributed based on previous analysis (Bressan et al. 2016).

Supplementary Figure S5

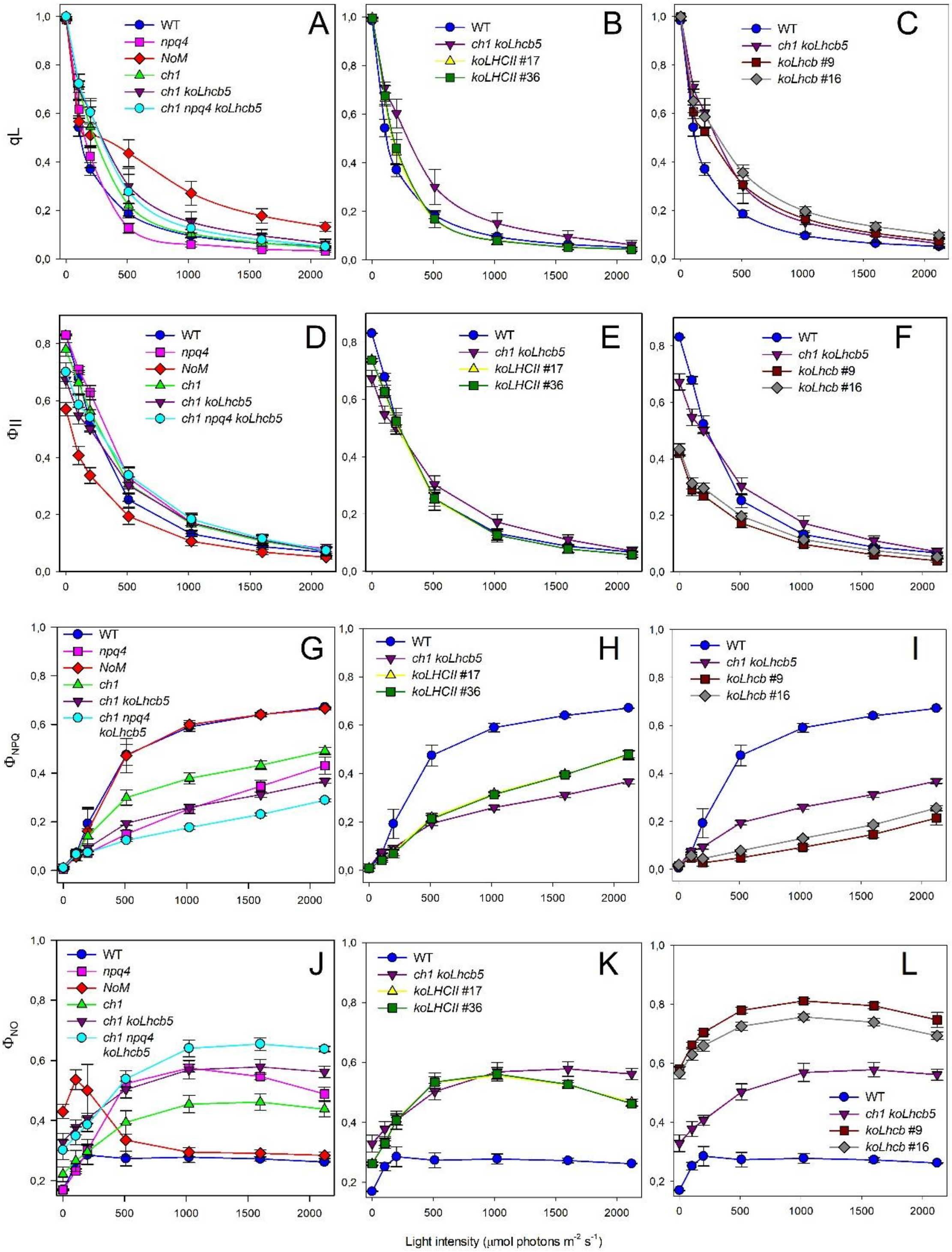

Supplementary Figure S5. Analysis of Chl fluorescence and energy partitioning during photosynthesis in wild type and mutant leaves at RT. (A-C) The light intensity dependence of the qL parameters reflects the redox state of the primary electron acceptor Q_A, indicating the fraction

of open PSII centers (with qL=0 meaning all centers are closed). (D-L) The light intensity dependence of various parameters is shown: quantum yield of PSII photochemistry (Φ_{II}, panels D-F), regulated quenching (Φ_{NPQ}, panels G-I) and unregulated quenching events (Φ_{NO}, panels J-L). Plants were dark-adapted for 30 min prior to the measurements. Each value is expressed as mean ± s.d., n = 5 independent plants.

Supplementary Figure S6

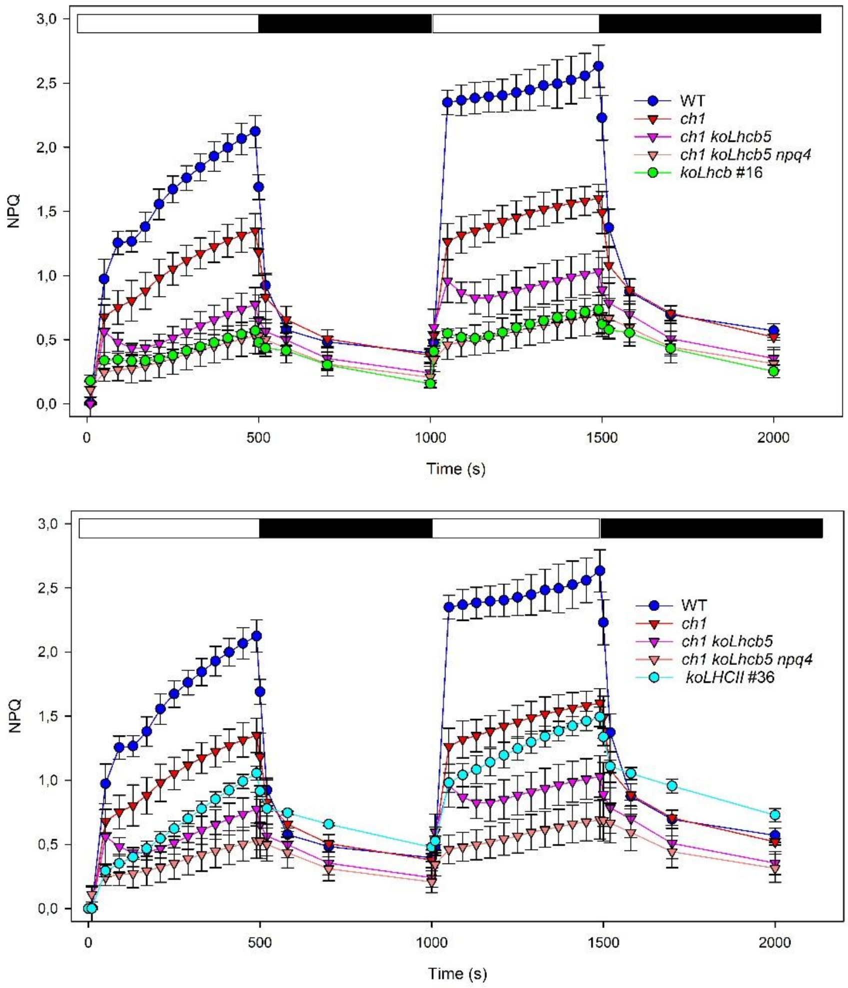

Supplementary Figure S6. Kinetics of formation and relaxation of photoprotective energy dissipation in wild type, *ch1,* and *LHC* mutant leaves. NPQ kinetics were measured in selected genotypes during two consecutive illumination periods with white light (1,000 μmol photons m^{-2} s^{-1}) at RT. White and black bars represent light and dark periods, respectively. Data are expressed as mean ± s.d., *n* = 4 biologically independent plants. The experiments were repeated independently twice, yielding similar results.

Supplementary Figure S7

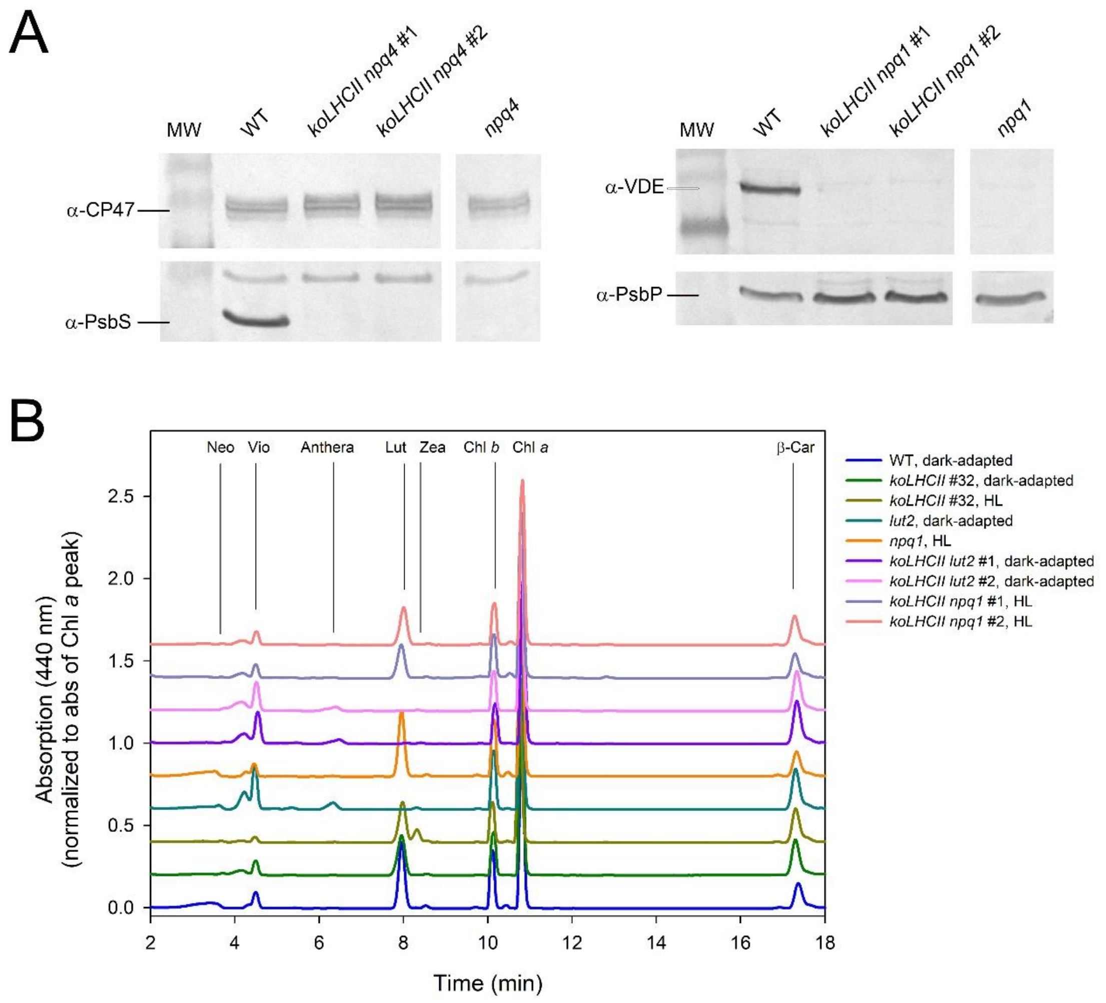

Supplementary Figure S7. Characterization of *koLHCII npq1* and *lut2* lines. Mutant lines were generated by combining *koLHCII* with either *npq1* or *lut2* single mutants through genome editing, followed by progeny selection. (A) Immunoblotting was performed to identify *koLHCII npq4* (left panel) and *koLHCII npq1* (right panel). The analysis used antibodies specific to the following gene products: VDE (violaxanthin de-epoxidase), PsbS, and the PSII core subunits PsbB (CP47) and PsbP. Leaf extracts containing approximately 2 µg of Chls were loaded for each sample, with all samples run on the same SDS–PAGE slab gel; the WT lane was used as the positive control, while the *npq4* and *npq1* lanes served as negative controls on the respective membranes. (B) Pigment content analysis of leaves from wild type and mutant plants. Lipid-soluble pigments were separated using HPLC. Each chromatogram represents the absorbance at 440 nm for pigments extracted from leaves either dark-adapted or exposed to HL (550 µmol photons m^{-2} s^{-1} at RT) for 20 min. Chromatograms were vertically shifted for easier comparison, and peaks corresponding to major

pigment species are indicated. The HPLC experiments were repeated independently twice, with similar results.

Supplementary Figure S8

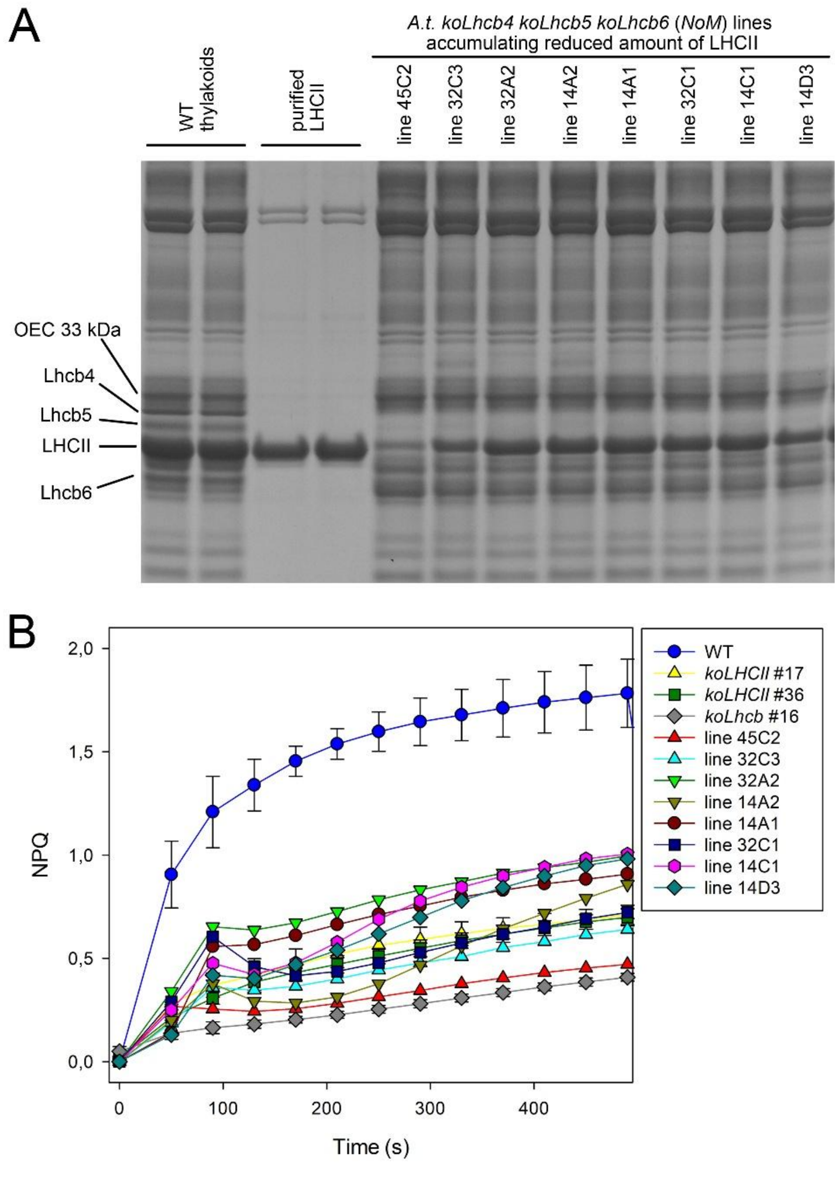

Supplementary Figure S8. Characterization of *NoM* lines expressing varying amounts of the LHCII antenna. (A) SDS–PAGE fractionation was used to analyze purified LHCII and thylakoid proteins from both WT and *NoM* lines with reduced LHCII levels. The latters were derived from a segregating population of *A. thaliana NoM* , generated by incomplete gene editing of both Lhcb1 and Lhcb2. For analysis, 4 µg of Chls from purified proteins and 10 µg of Chls from thylakoids were loaded. LHCII content (mol protein / mol Chls in thylakoids) was quantified by densitometric analysis

of the Coomassie-stained gels. (B) The kinetics of photoprotective energy dissipation formation and relaxation were measured after illuminating leaves with 1,000 μmol photons m^{-2} s^{-1} at RT. Data for WT, *koLHCII* #17 and #36, *koLhcb* #16 lines are expressed as mean ± s.d., *n* = 4 biologically independent plants. For the segregating population, each trace represents and individual plant. The experiments were independently repeated twice, with consistent results.

Supplementary Figure S9

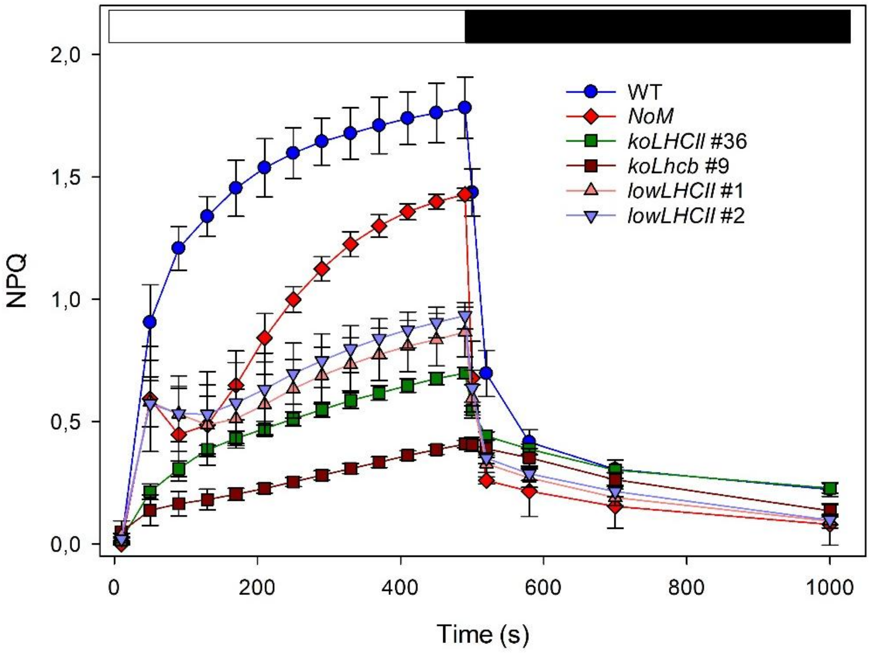

Supplementary Figure S9. Photoprotective energy dissipation of *lowLHCII* lines. The kinetics of photoprotective energy dissipation formation and relaxation were measured after illuminating leaves with 1,000 μmol photons m^{-2} s^{-1} at RT. White and black bars represent light and dark phases, respectively. Data are presented as mean ± s.d., *n* = 4 biologically independent plants. The experiments were repeated twice independently, yielding consistent results.

Supplementary Figure S10

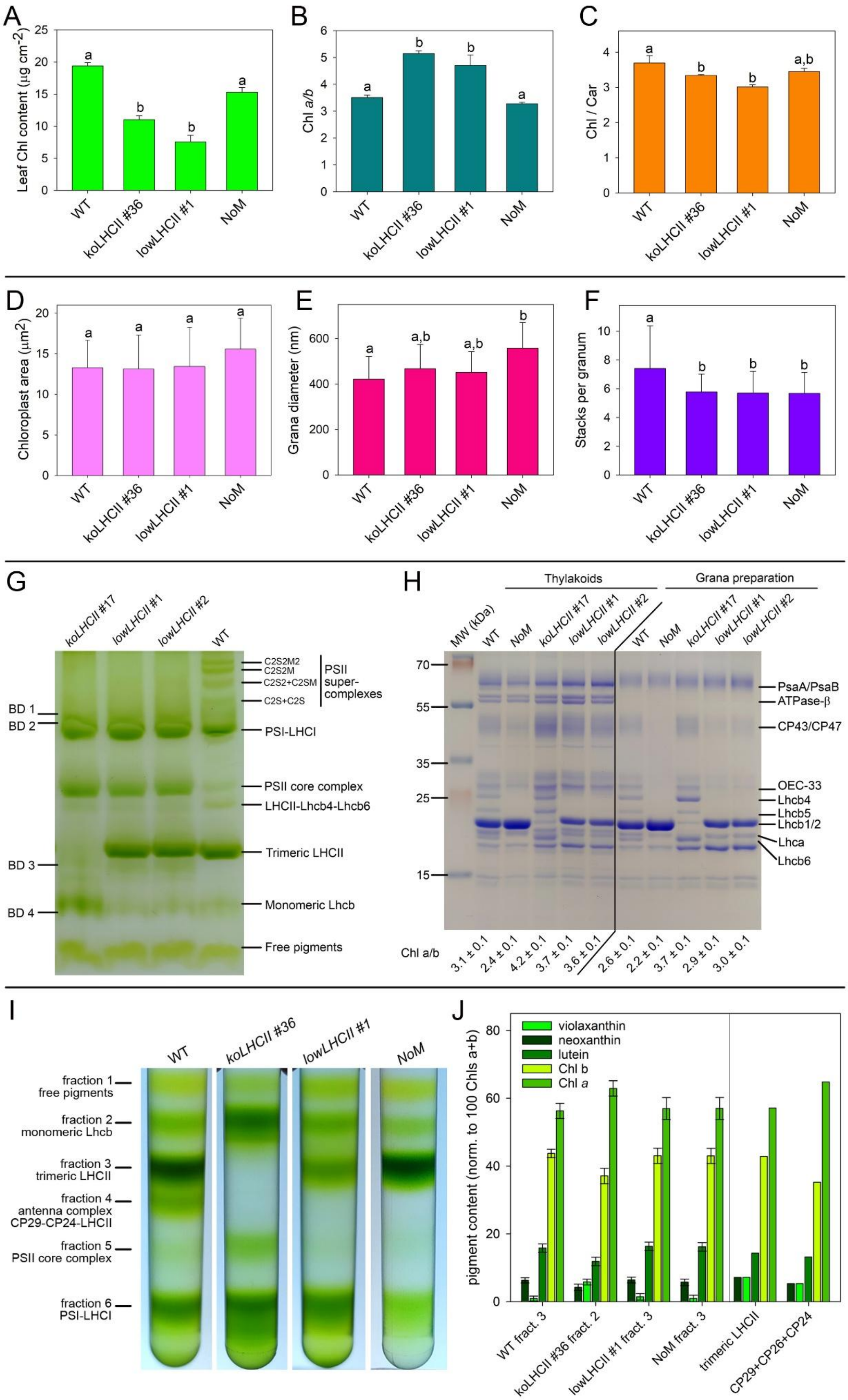

Supplementary Figure S10. Characterization of *koLHCII*, *lowLHCII* and *NoM* mutant plants. (A-C) Pigment content was determined for leaves of WT and mutant lines, with at least six different plants tested for each line. The Chl/Car ratio represents the molar ratio between chlorophylls (Chl *a* + *b*) and carotenoids. The pigment content in leaves, indicative of the abundance of pigment-protein complexes, was similar between *koLHCII* and *lowLHCII* plants. (D-E) Transmission electron micrographs of leaf mesophyll tissue were analyzed to assess plastids characteristics. The histograms display the following: (D) chloroplast area [n ≥ 10]; (E) grana diameter [n = 60]; (F) the number of stacks per granum [(n ≥ 30]**.** The chloroplasts were essentially the same size across all genotypes, while the number of grana stacks was similarly reduced in both *koLHCII* and *lowLHCII* compared to the WT. (G) Non-denaturing Deriphat-PAGE of thylakoids solubilized with 0.8% α-DM. Each lane contained thylakoid proteins corresponding to 35 μg of Chl. The composition of the major bands is indicated as in Figure 1B. The relative abundance of PSI and PSII was comparable among these genotypes. (H) Membranes enriched in grana partitions were isolated following the method from (Morosinotto et al. 2010), we then evaluated polypeptide composition and assessed Chl content, see SDS-PAGE profiles of thylakoids (*left*) and grana preparation (*right*). Major polypeptide bands and molecular weights reference (MW) are indicated, Chl a/b ratios are reported above. In the grana-enriched preparations from both *koLHCII* and *lowLHCII*, ATPase subunits were no longer detectable, and PSI core subunits were depleted compared to intact thylakoids. Additionally, the Chl *a* / *b* ratio decreases similarly in both genotypes. (I) Thylakoid membranes isolated from WT and mutant plants were solubilized using 1.0 % α-DM, followed by the fractionation of pigment-protein complexes through sucrose gradients ultracentrifugation. The composition of the main green bands is indicated. (J) For each gradient, green fractions were collected, and the contents of Chl and xanthophylls were assessed by HPLC, normalized to 100 Chls *a* + *b*. The right panel presents the pigment composition of trimeric LHCII and monomeric Lhcb (Su et al. 2017) for reference. Notably, all fraction 3 samples are depleted of violaxanthin; since this xanthophyll is bound to the external site V1, its low abundance is likely due to detachment during thylakoid solubilization. In conclusion, fractionation of thylakoid membranes showed that Chl and xanthophylls were similarly associated with pigment-binding proteins in mutant lines as in the WT, without the accumulation of “free Chls” in the membranes which, if occurring, could have induced oxidative stress (Havaux et al. 2007). Data are shown as mean ± SD, n=3. Values that are significantly different (ANOVA followed by Tukey's post hoc test at a significance level of $P < 0.05$) are marked with different letters. Densitometric analysis of the green fractions revealed no significant differences in the levels of free Chls between WT and mutant lines (data not shown).

Supplementary Figure S11

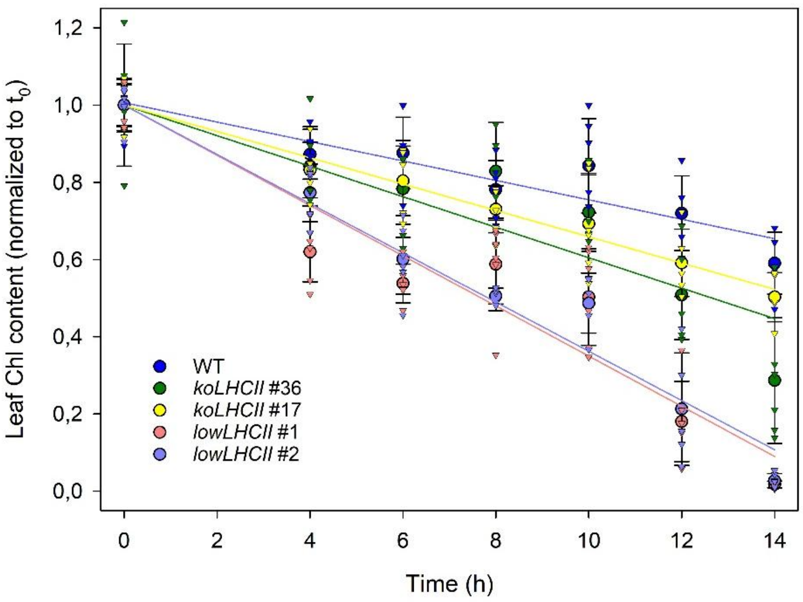

Supplementary Figure S11. Photoprotective role of LHC. Leaf discs placed on moist paper were exposed to 1,800 µmol photons m^{-2} s^{-1} at 4°C, and the kinetics of chlorophyll bleaching were monitored. The data were modeled using linear regression analysis. Statistical testing (F-test) revealed that photobleaching occurred more rapidly in both *lowLHCII* leaves, which showed significantly higher photosensitivity compared to other genotypes. Specifically, the differences between the slopes of the *koLHCII* mutants and *lowLHCII* leaves were statistically significant ($P < 0.05$). Results are expressed as mean ± standard deviation (circles), with n = 5 biologically independent plants, and individual data points are also shown (triangles).

Supplementary Figure S12

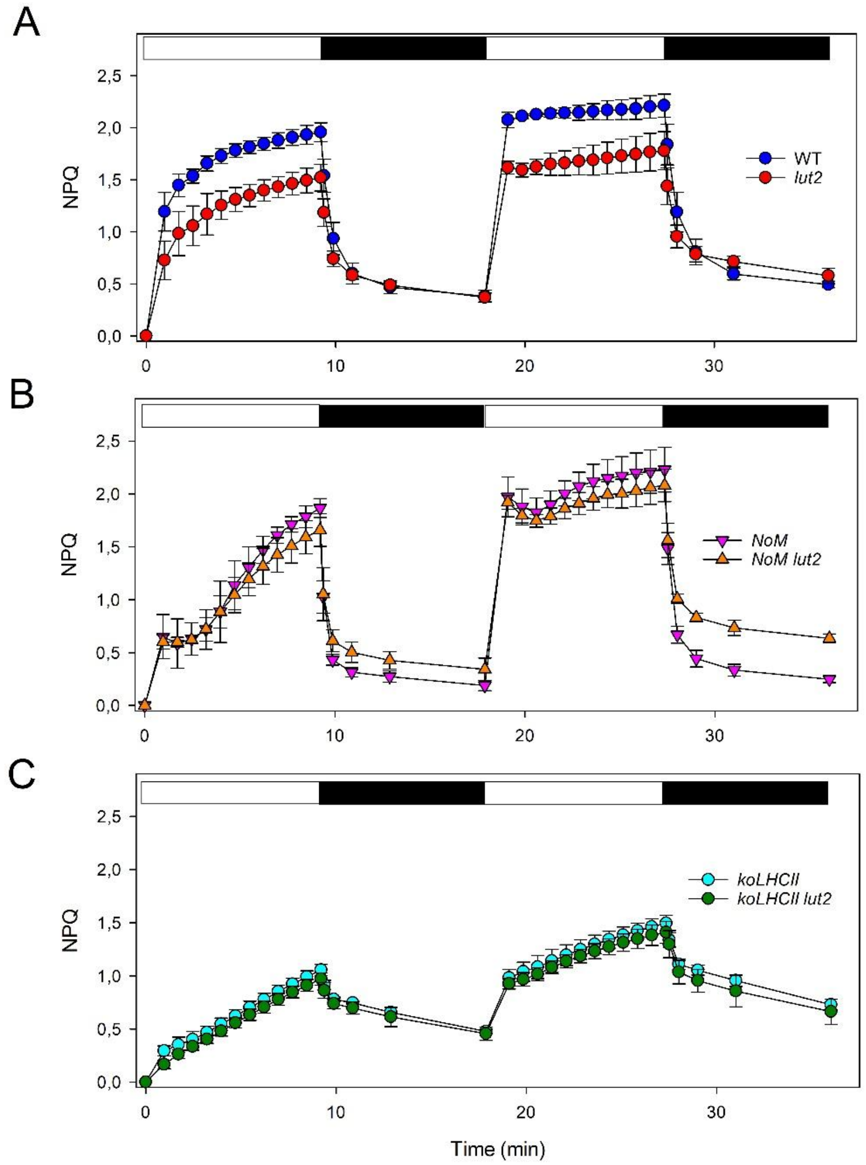

Supplementary Figure S12. **Kinetics of formation and relaxation of photoprotective energy dissipation in wild type and mutants *NoM, koLHCII and lut2***. NPQ kinetics were measured in leaves of WT and *lut2* (C), *NoM* and *NoM lut2* (B), *koLHCII* and *koLHCII lut2* (C), during two consecutive periods of illumination with white light (1,000 µmol photons m^{-2} s^{-1}) at RT. White and black bars represent light and dark periods, respectively. Data are expressed as mean ± s.d., *n* = 4 biologically independent plants. The experiments were repeated independently twice, producing similar results.

Supplementary Table S1

Target	sgRNA
Lhcb1.1 - Lhcb1.5	GGTTCACAGATCTTCAGCGA<br>ATGGACCC AAGTACTTGACT<br>TGTGGATAACTTCT AGCTCA<br>GGCTACTCAAGTTATCCTCA<br>GAAGCGGCCGTGTGACAATG<br>AGAAGTTATCCACAGCAGGT<br>GAGGACTTGCTTTACCCCGG<br>AGGGGAGGAGAGAGCCATTG
Lhcb2.1 - Lhcb2.3	CGCAAGGTTGGTGTATCCGG<br>TGGAGGGCTTGAGAGCCGTT<br>CGCCCAAGACGCCCACCTTT<br>AGACTTGACGGTACGACGCA<br>AGGGCTTGACCCGCTTTACC<br>GGATCAAGTTAGGGTTTCCG<br>CCAACATTGCCCATCTACTG<br>TCTGGGCTGTTCAAGTTGTG
Psbs	GTTCCTCAAAAAATGGCCGG<br>GAACTTTGGGCTGAGCCAAA<br>CTTACTTCAGGCGTCACCGC<br>CTCCGGTTGCACTACCATCT
**carotene ε-cyclase (ε-CYC)**	TGTAGTGTCAGAGCTAGCGG<br>CGGTAGTGAGAGTTGTGTAG<br>TTCCTTCGACAACTCCATGA<br>TGTAGTGTCAGAGCTAGCGG
**violaxanthin de-epoxidase (VDE)**	AATCGAATACGGTCATGACA<br>GCTGATCTCAGAACAACTGG<br>CGACATTGGCTGCACAGGCA<br>CATCTTGTATCCACCCACGA

Supplementary Table S1. Target sites used for sgRNA design. The sequences designed for the Lhcb1 genes were identical to those reported by (Ordon et al. 2020).

Supplementary Table S2

	+ DCMU	- DCMU
	F_v / F_m	F_v / F_m
**WT**	0.756 ± 0.008^{a}	0.820 ± 0.006^{a}
koLHCII	0.624 ± 0.014^{b}	0.761 ± 0.007^{b}
NoM	0.388 ± 0.054^{c}	0.615 ± 0.007^{c}
koLhcb	0.184 ± 0.109^{d}	0.505 ± 0.024^{d}

Supplementary Table S2. F_v / F_m values determined in leaves from wild type and mutant plants. The quantum yield of PSII was measured in dark-adapted leaves, both before and after vacuum-infiltration with $3 \cdot 10^{-5}$ M DCMU. Data are expressed as mean ± s.d., *n* = 5 biologically independent plants. Values marked with different letters indicate significant differences within the column (ANOVA, followed by Tukey's post-hoc test at a significance level of $P < 0.05$).

Supplementary Table S3

	VAZ (μmol / 100 μmol Chls)
WT	5.2 ± 0.1[a]
koLHCII	7.6 ± 0.5[b]
koLhcb	9.4 ± 2.5[c]

Supplementary Table S3. Total VAZ (violaxanthin + antheraxanthin + zeaxanthin) content in leaves from wild type and mutant lines. Pigments were extracted from dark-adapted leaves using 85% acetone, then separated and quantified via HPLC. Data are normalized to 100 Chl *a* + *b* molecules and are presented as mean ± s.d., n = 3 biologically independent samples. Values marked with different letters indicate significant differences (ANOVA followed by Tukey's post-hoc test at a significance level of $P < 0.05$). The results shown are representative of the independent lines.

Supplementary Table S4

	Chl a/b	Chl/Car	µg Chl / cm^2
WT	3.08 ± 0.08[a]	4.32 ± 0.10[a]	24.8 ± 1.7[a]
npq1	3.07 ± 0.07[a]	4.41 ± 0.11[a]	28.4 ± 1.3[b]
lut2	3.19 ± 0.08[a]	5.22 ± 0.37[b]	24.8 ± 2.2[a]
koLHCII #36	4.07 ± 0.04[b,c]	4.63 ± 0.35[a,b]	14.0 ± 1.5[c]
koLHCII npq1 #1	3.88 ± 0.19[b]	4.28 ± 0.21[a]	11.2 ± 1.4[c]
koLHCII lut2 #1	4.18 ± 0.18[c]	4.62 ± 0.83[a,b]	12.3 ± 1.5[c]

Supplementary Table S4. Pigment content determination for wild type, *koLHCII*, *npq1*, and *lut2* lines. Parameter content was measured in T3 generation *koLHCII npq1* and *koLHCII lut2* plants. The Chl/Car ratio represents the molar ratio between chlorophylls (*a* + *b*) and carotenoids. Leaf Chl content was measured after 6 weeks of growth under control conditions. Data are expressed as mean ± s.d., n = 5 biologically independent leaves. Values marked with different letters indicate significant differences within the column (ANOVA followed by Tukey's post-hoc test at a significance level of $P < 0.05$). The results displayed are representative of the independent lines.

Supplementary Table S5

Genotype	Missing protein(s)	Phenotype	Chl *a/b* ratio	Leaf Chl content (μg cm^{-2})	qE	Fv / Fm	Characterized by
wild type (WT)	-	-	3.5	19.4	1.73 ± 0.1	0.82	-
koLHCII #17 / #36	Lhcb1, Lhcb2, Lhcb3	devoid of trimeric LHCII	5.2	11.2	0.55 ± 0.1	0.76	this work
koLhcb #9 / #16	Lhcb1, Lhcb2, Lhcb3, Lhcb4, Lhcb5, Lhcb6	devoid of PSII outer antenna system	6.2	6.1	0.33 ± 0.1	0.51	this work
NoM	Lhcb4, Lhcb5, Lhcb6	devoid of PSII monomeric antennae	3.3*	15.3	1.67 ± 0.1	0.62	(Dall'Osto et al. 2017)
npq4	PsbS	defective in qE	3.4*	21.1	0.20 ± 0.1	0.82	(Li et al. 2000)
npq1	violaxanthin de-epoxidase (VDE)	defective in the xanthophyll cycle	*	-	0.58 ± 0.1	0.82	(Niyogi et al. 2001)
lut2	carotene ε-cyclase (ε-CYC)	defective in the lutein biosynthesis	*	-	1.03 ± 0.1	0.82	(Niyogi et al. 2001; Lokstein et al. 2002)
koLHCII npq4	Lhcb1, Lhcb2, Lhcb3, PsbS	devoid of both trimeric LHCII and PsbS	-	-	0.21 ± 0.1	0.76	this work
koLHCII npq1	Lhcb1, Lhcb2, Lhcb3, VDE	devoid of both trimeric LHCII and zeaxanthin	**	-	0.27 ± 0.1	0.76	this work
koLHCII lut2	Lhcb1, Lhcb2, Lhcb3, ε-CYC	devoid of both trimeric LHCII and lutein	**	-	0.52 ± 0.1	0.76	this work
lowLHCII #1 / #2	Lhcb3, lower expression of Lhcb1 and Lhcb2	express one trimeric LHCII per PSII core complex	**	7.4	0.80 ± 0.1	0.55	this work
ch1	chlorophyll *a* oxygenase (CAO)	defective in Chl *b* biosynthesis, impaired accumulation of Lhcb except for the overexpressed Lhcb5	-	6.6	0.97 ± 0.1	0.78	(Havaux et al. 2007)
ch1 koLhcb5	CAO, Lhcb5	defective in Chl *b* biosynthesis, impaired accumulation of all PSII antennae	-	4.5	0.53 ± 0.2	0.67	(Havaux et al. 2007)
ch1 koLhcb5 npq4	CAO, Lhcb5, PsbS	defective in Chl *b* biosynthesis, devoid of both PSII outer antenna system and PsbS	-	3.9	0.32 ± 0.2	0.67	this work

***not statistically different (respect to WT)**

****not statistically different (respect to *koLHCII*)**

Supplementary Table S5. Table summarizing the *Arabidopsis* mutants used in this study.

Supplementary References

Bressan M, Dall'Osto L, Bargigia I, Alcocer MJP, Viola D, Cerullo G, D'Andrea C, Bassi R, Ballottari M. LHCII can substitute for LHCI as an antenna for photosystem i but with reduced light-harvesting capacity. *Nat Plants*. 2016:2:1–10. https://doi.org/10.1038/nplants.2016.131.

Dall'Osto L, Cazzaniga S, Bressan M, Paleček D, Židek K, Niyogi KK, Fleming GR, Zigmantas D, Bassi R. Two mechanisms for dissipation of excess light in monomeric and trimeric light-harvesting complexes. *Nat Plants*. 2017:3:17033. https://doi.org/10.1038/nplants.2017.33.

Havaux M, Dall'Osto L, Bassi R. Zeaxanthin has enhanced antioxidant capacity with respect to all other xanthophylls in arabidopsis leaves and functions independent of binding to PSII antennae. *Plant Physiol*. 2007:145:1506–1520. https://doi.org/10.1104/pp.107.108480.

Laemmli UK. Cleavage of structural proteins during the assembly of the head of bacteriophage T4.

Morosinotto T, Segalla A, Giacometti GM, Bassi R. Purification of structurally intact grana from plants thylakoids membranes. *J Bioenerg Biomembr*. 2010:42:37–45. https://doi.org/10.1007/s10863-009-9261-3.

Li XP, Björkman O, Shih C, Grossman AR, Rosenquist M, Jansson S, Niyogi KK. A pigment-binding protein essential for regulation of photosynthetic light harvesting. *Nature*. 2000:403:391–395. https://doi.org/10.1038/35000131.

Lokstein H, Tian L, Polle JE, DellaPenna D. Xanthophyll biosynthetic mutants of Arabidopsis thaliana: altered nonphotochemical quenching of chlorophyll fluorescence is due to changes in Photosystem II antenna size and stability. *BiochimBiophysActa*. 2002:1553:309–319. https://doi.org/10.1016/s0005-2728(02)00184-6.

Niyogi K, Shih C, Soon Chow W, Pogson B, DellaPenna D, Björkman O. Photoprotection in a zeaxanthin- and lutein-deficient double mutant of Arabidopsis. *Photosynth Res*. 2001:67:139–145. https://doi.org/10.1023/A:1010661102365.

Ordon J, Bressan M, Kretschmer C, Dall'Osto L, Marillonnet S, Bassi R, Stuttmann J. Optimized Cas9 expression systems for highly efficient Arabidopsis genome editing facilitate isolation of complex alleles in a single generation. *Funct Integr Genomics*. 2020:20:151–162. https://doi.org/10.1007/s10142-019-00665-4.

Schägger H, von Jagow G**.** Tricine-sodium dodecyl sulfate-polyacrylamide gel electrophoresis for the separation of proteins in the range from 1 to 100 kDa. *AnalBiochem*. 1987:166:368–379. https://doi.org/10.1016/0003-2697(87)90587-2.

Su X, Ma J, Wei X, Cao P, Zhu D, Chang W, Liu Z, Zhang X, Li M, Ma J, et al**.** Structure and assembly mechanism of plant C2S2M2-type PSII-LHCII supercomplex. *Science* . 2017:357:815–820. https://doi.org/10.1126/science.aan0327.
